# Optimal dosage and modality of exercise on glycemic control in people with prediabetes: a systematic review and network meta-analysis

**DOI:** 10.3389/fendo.2025.1560676

**Published:** 2025-04-28

**Authors:** Lin Zhang, Xing Cheng, Yong Yang, Xue Li, Yuan Yuan

**Affiliations:** ^1^ Department of Rehabilitation, Jintang First People’s Hospital, Chengdu, China; ^2^ College of Sports Medicine and Health, Chengdu Sport University, Chengdu, China; ^3^ Laboratory of Kinesiology and Rehabilitation, School of Physical Education and Sport, Chaohu University, Hefei, China; ^4^ The Rehabilitation Medicine Center, People’s Hospital of Deyang City, Deyang, China; ^5^ Department of Sport and Exercise Sciences, Kunsan National University, Gunsan-Si, Republic of Korea

**Keywords:** exercise, dose-response relationship, prediabetes, glycemic control, resistance training

## Abstract

**Aims:**

This study aims to assess the effects of different exercise types and their specific doses on glycemic control among individuals with prediabetes.

**Methods:**

Multiple databases were subjected to a comprehensive search for randomized controlled trials (RCTs) published until 15 July 2024. The study protocol was prospectively registered with PROSPERO (CRD42024573186). The exercise interventions analyzed included aerobic exercise (AE), resistance training (RT), and combined aerobic–resistance training (AE+RT). Outcomes were quantified using standardized mean difference (SMD) with 95% credible intervals (CrIs), employing the confidence in network meta-analysis (CINeMA) framework for network meta-analysis to confirm the outcome reliability.

**Results:**

According to the network meta-analysis, irrespective of dose, AE+RT led to the largest decrease in fasting blood glucose (FBG) (−0.44, [−0.62 to −0.26]). AE alone resulted in the largest reductions in 2-hour post-meal blood glucose (2hPG) (−0.71, [−0.97 to −0.45]) and glycosylated hemoglobin A1c (HbA1c) (−0.30, [−0.37 to −0.22]). Dose–response (DR) analysis identified optimal doses for each exercise type: 880 metabolic equivalent of task minutes per week (METs-min/week) for both AE and RT and 800 METs-min/week for AE+RT to reduce FBG. The optimal dose for 2hPG improvement via AE was 1,100 METs-min/week, and for HbA1c reduction via RT, it was 870 METs-min/week.

**Conclusions:**

Given the variety of impaired glucose regulation (IGR), we recommend that people with prediabetes engage in RT at 1,100 METs-min/week to improve 2hPG and at 870 METs-min/week to reduce HbA1c. For FBG control, a dose of 800 METs-min/week is optimal for all exercise modalities. These evidence-based recommendations provide practical guidance for designing personalized exercise prescriptions to manage prediabetes.

## Background

Prediabetes is an intermediate stage of glucose dysregulation that can progress to type 2 diabetes (T2D) if untreated and characterized by higher levels of glycosylated hemoglobin A1c (HbA1c), 2-hour post-meal blood glucose (2hPG), or fasting blood glucose (FBG), which exceed normal values without meeting the diagnostic criteria for diabetes (1, 2). Nearly 1/3 of the Chinese population suffers from prediabetes, a rate similar to global figures, including those in the United States (3, 4). Annually, approximately 5% to 10% of individuals with prediabetes develop T2D. In response, the American Diabetes Association recommends promoting healthy lifestyles, especially physical activity, to manage or reverse prediabetes and prevent its escalation.

Regular physical activity has proven effective in enhancing glucose regulation and substantially lowering the risk of T2D, potentially delaying or averting its onset. Studies have consistently supported moderate-intensity exercise as crucial in reducing diabetes risk, with recommended levels of 2.5 hours per week, or 0.5 hours daily over 5 days (5–10). Although numerous meta-analyses have assessed the impacts of various physical activities on health outcomes, none have pinpointed the optimal exercise type and dose to substantiate these guidelines, leaving uncertainty about the adequacy of physical activity levels (11–17).

Bayesian dose–response (DR) meta-analysis models have successfully identified optimal types and doses of physical activity for certain outcomes, including HbA1c levels, thus offering insights into the best exercise combinations for glycemic control in prediabetes—a persistently unanswered question. Moreover, the influence of critical clinical factors, such as baseline FBG and 2hPG levels, on the effectiveness of physical activity programs remains unexplored, which hinders the creation of tailored physical activity recommendations.

This study utilizes a meta-analysis framed within a Bayesian context, drawing on data from established randomized controlled trials (RCTs) to assess the efficacy of aerobic exercise (AE), resistance training (RT), and combined aerobic–resistance training (AE+RT) in lowering FBG, 2hPG, and HbA1c in individuals suffering from prediabetes. Furthermore, it engages in examining the dose–response relationship (DRR) between the above exercise forms and glycemic regulation. For the enhancement of clinical applicability, the findings are translated into practical exercise prescriptions, such as 900 METs-min/week of AE, which equates to four 40-minute sessions of low-impact aerobic dancing weekly (code: 03020).

## Methods

This preregistered systematic review and network meta-analysis obeyed the Preferred Reporting Items for Systematic Reviews and Meta-Analyses (PRISMA) checklist guidelines (PROSPERO registration no. CRD42024573186) (18).

### Data sources and search strategy

Without linguistic limitations, a comprehensive search was executed from the beginning of their existence until July 15, 2024, throughout Medline, PubMed, Sport Discus, WOS, and the Cochrane Library, with the search strategy designed using a combination of Medical Subject Headings (MeSH) terms and free-text keywords related to prediabetes, exercise, and RCTs; readers are allowed to find the whole search plan in the first **Supplementary File**. Additional research was also retrieved by reviewing the reference lists of pertinent publications and reviews. Two researchers (LZ and XC) took charge of double-screening titles, abstracts, and complete texts, and a third author (YY) was introduced to handle any possible disagreements.

### Study selection and data extraction

As described in **Table 1**, the authors LZ and XC worked separately to include RCTs covering adult participants (age ≥ 18 years) with a diagnosis of prediabetes according to the American Diabetes Association (ADA), World Health Organization (WHO), or International Expert Committee criteria, without any restrictions on gender, region, race, socioeconomic status, or severity of prediabetes. Studies must evaluate any form of exercise therapy (AE or RT) as a single or combined intervention and compare it with another exercise therapy, waiting lists, regular daily activities, usual care, health education, or equivalent control interventions. Studies that failed to give a clear definition of the type or dose of exercise and those that did not report required outcome measures were excluded. Any differences that arose were then resolved through discussion among the authors. As stated in Section 7.7.3 of the Cochrane Handbook for Systematic Evaluation of Interventions, the formula was used for the calculation of the standard deviation (SD) in cases where it was not present, SD = SE × √n, or derived from t-values, interquartile ranges, confidence intervals, or p-values. If data were unobtainable, the corresponding author was contacted. Only pre- and post-intervention data were merged to minimize Type I errors.

**Table 1:** Selection criteria.

Category	Inclusion criteria	Exclusion criteria
Population	-Adults older than 18 years with prediabetes. -Prediabetes diagnosed by the ADA, WHO, or the International Expert Committee. -All genders, ethnicities, and severity of prediabetes to be included.	Adults without a definite prediabetes diagnosis at baseline.
Intervention	-Any type of exercise including aerobic exercise, resistance training, and combined intervention.	Any exercise training was not clearly defined in terms of type or dosage.
Comparator	-Waiting list, regular daily activities, usual care, health education, and equivalent control intervention. -Another type of exercise to facilitate direct comparisons.	No suitable control or unclear description of the control group.
Outcome	At least one measure of FBG, 2hPG, and HbA1c.	Acute effects of a single session.
Study design	RCTs (individual design, cluster design, or the first half of crossover).	Non-randomized controlled trial (reviews, case reports, etc.).

ADA, American Diabetes Association; WHO, World Health Organization; FBG, fasting blood glucose; 2hPG, 2-hour post-meal blood glucose; HbA1c, glycosylated hemoglobin A1c; RCTs, randomized controlled trials.

### Risk of bias and quality of evidence assessment

Three writers—LZ, XC, and XL—adopted the Cochrane Risk of Bias instrument for the independent evaluation of the potential for bias (19), involving six main sections: random sequence generation, allocation concealment, blinding of participants and researchers, blinding of outcome assessment, incomplete outcome data, and selective reporting. Another technique for evidence assessment was confidence in network meta-analysis (CINeMA), a web-based program that sorts confidence levels into four categories: high, moderate, low, and extremely low (20).

### Data coding and management

Extensive data coding was used to investigate the DR connection between exercise and glycemic control in prediabetes people. The interventions first fell into “Exercise” or “Con” (control). Afterward, the workouts were sorted into the AE, RT, and AE+RT groups according to the mode of energy delivery. “Exercise dose” refers to energy expenditure assessed in metabolic equivalent of task minutes per week (METs-min/week), where 1 metabolic equivalent of task (MET) represents the resting metabolic rate (3.5 mL O^2^/kg/min). Total weekly METs-min was calculated as follows: MET value of activity × duration per session (minutes) × sessions per week (21). Interventions were then categorized according to their specific type and dosage. For enhanced network connectivity, approximate values were assigned to exercise doses as 0 (Con), 250, 500, 750, 1,000, or 1,250 METs-min/week, a method utilized in previous studies (22–24).

### Data synthesis and analysis

Statistical analysis relied on Stata (version 17.0) and R (version 4.3.2) (25, 26). In Stata, network plots generated through the “network map” command the viability of conducting a network meta-analysis. The network meta-analysis utilized the netmeta package within a frequentist framework, facilitating comparisons of exercise types on glycemic control and generating league tables for FBG, 2hPG, and HbA1c. As the data involved continuous outcomes, they were assessed using standardized mean difference (SMD) with 95% credible intervals (CrIs). A random effects model was applied for data synthesis, employing P-scores from the surface under the cumulative ranking curve (SUCRA) for ranking treatment efficacy. The R decomp.design function evaluated global heterogeneity (I^2^) and inconsistency; an I^2^ value over 50% indicated significant heterogeneity, and p < 0.05 suggested notable inconsistency overall. Local inconsistency was examined through the separating indirect from direct evidence (SIDE) test (27). Meta-regression was conducted using gemtc to identify potential heterogeneity sources and to refine the robustness of network meta-analysis results by centering values. To detect publication bias, comparison-corrected funnel plots and Egger’s test were used, and p < 0.05 denoted significant bias.

Additionally, analysis of the DRRs was conducted via the MBNMAdose package in R (28). Treatment-level and agent-level network plots confirmed connectivity (**Supplementary File 10**,**Figure 10.1.1~10.1.3**), and both unrelated mean effect and consistency models were applied to analyze the data, comparing estimated parameters, model deviations, and deviance information criterion (DIC) scores for robustness (**Supplementary File 10**,**Table 10.3**) (29). The node-splitting approach of MBNMA was employed to verify transitivity, with similarity in effects demonstrating good correspondence between direct and indirect evidence (**Supplementary File 10**,**Table 10.4**) (30). Restricted cubic splines were utilized to determine the best-fitting DRR among various non-linear models (31). Knots were placed at the 10th, 50th, and 90th percentiles of treatment doses to optimize model fit and biological relevance, and a Wald test assessed deviations from linearity (32).

## Results

**Figure 1:**
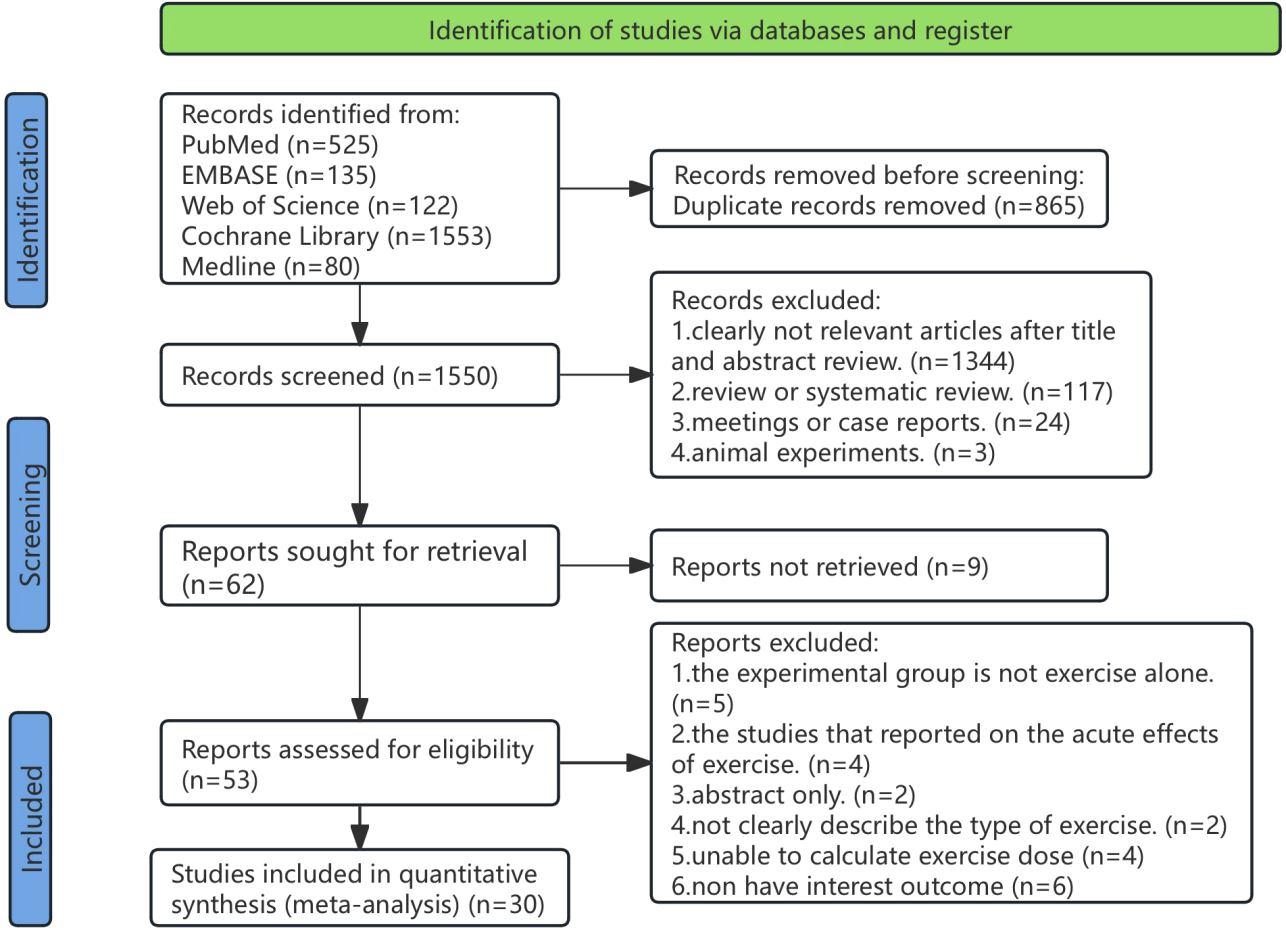
PRISMA flow diagram illustrating the search process for studies to identify RCTs. RCTs, randomized controlled trials.

An initial electronic search yielded 2,449 records; after removing duplicates, 1,584 records underwent title and abstract screening. Ultimately, 56 full-text articles underwent eligibility assessment, resulting in 30 studies (**Supplementary References 1–30**) being included in this review, encompassing 2,895 participants (1,606 and 1,289 in the treatment group and the control group, respectively) (**Figure 1**).

### Characteristics of included studies

Study characteristics are detailed in **Supplementary File 2**, where 1,048 participants from 28 studies participated in AE, 406 in RT from 13 studies, and 110 in AE+RT from six studies. The sample sizes ranged from 7 to 136, with a median of 72, and participant ages ranged from 33.9 to 72.25 years, with a median age of 53.07. These studies spanned from 1998 to 2023, with a median publication year of 2010. The median duration of exercise interventions was 78 weeks (ranging from 12 to 144 weeks), typically involving three sessions per week (ranging from 2 to 5 sessions) lasting an average of 52 minutes per session (ranging from 14 to 90 minutes).

### Risk of bias and quality of evidence

**Figure 2:**
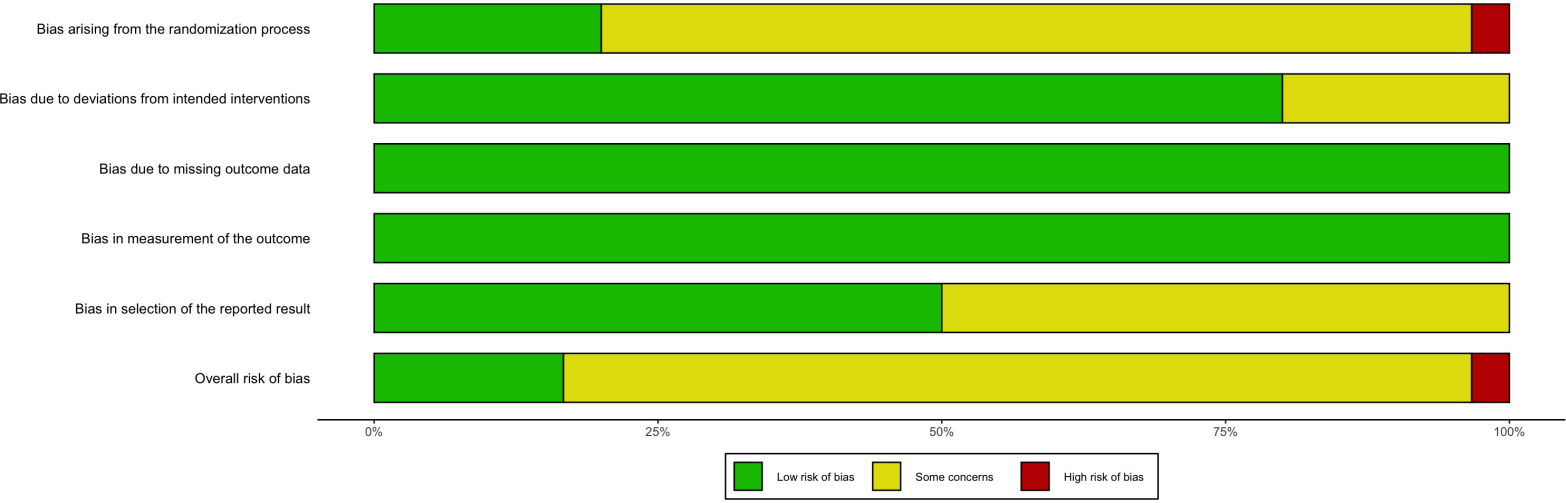
Risk of Bias version 2 summary.

Among the reviewed studies, 24 (80%) exhibited some level of bias, one (3.33%) exhibited a high risk of bias, and five (16.67%) exhibited a low risk of bias (**Supplementary Table 3.1**, **Figure 2**). Primary bias sources included 1) challenges in blinding participants and personnel resulting from the nature of the interventions and 2) inadequate reporting of key methodological aspects like random sequence generation, contributing to an ambiguous risk of bias. Following the CINeMA evaluation, the evidence quality was deemed low.

### Network meta-analysis and DRR

#### FBG

**Supplementary Figure 4.1.1** illustrates the direct comparisons and sample size distributions among different exercise modalities. In the forest and league plots, it was noted that AE+RT (SMD, −0.44; 95% CrIs [−0.62 to −0.26]), RT (SMD, −0.43; 95% CrIs [−0.57 to −0.28]), and AE (SMD, −0.40; 95% CrIs [−0.51 to −0.29]) showed higher effectiveness in FBG reduction versus the Con. AE was particularly more effective than the other exercise types (SUCRA, 0.81) (**Supplementary Figure 4.2.1**, **Supplementary Table 4.1.2**).

**Figure 3:**
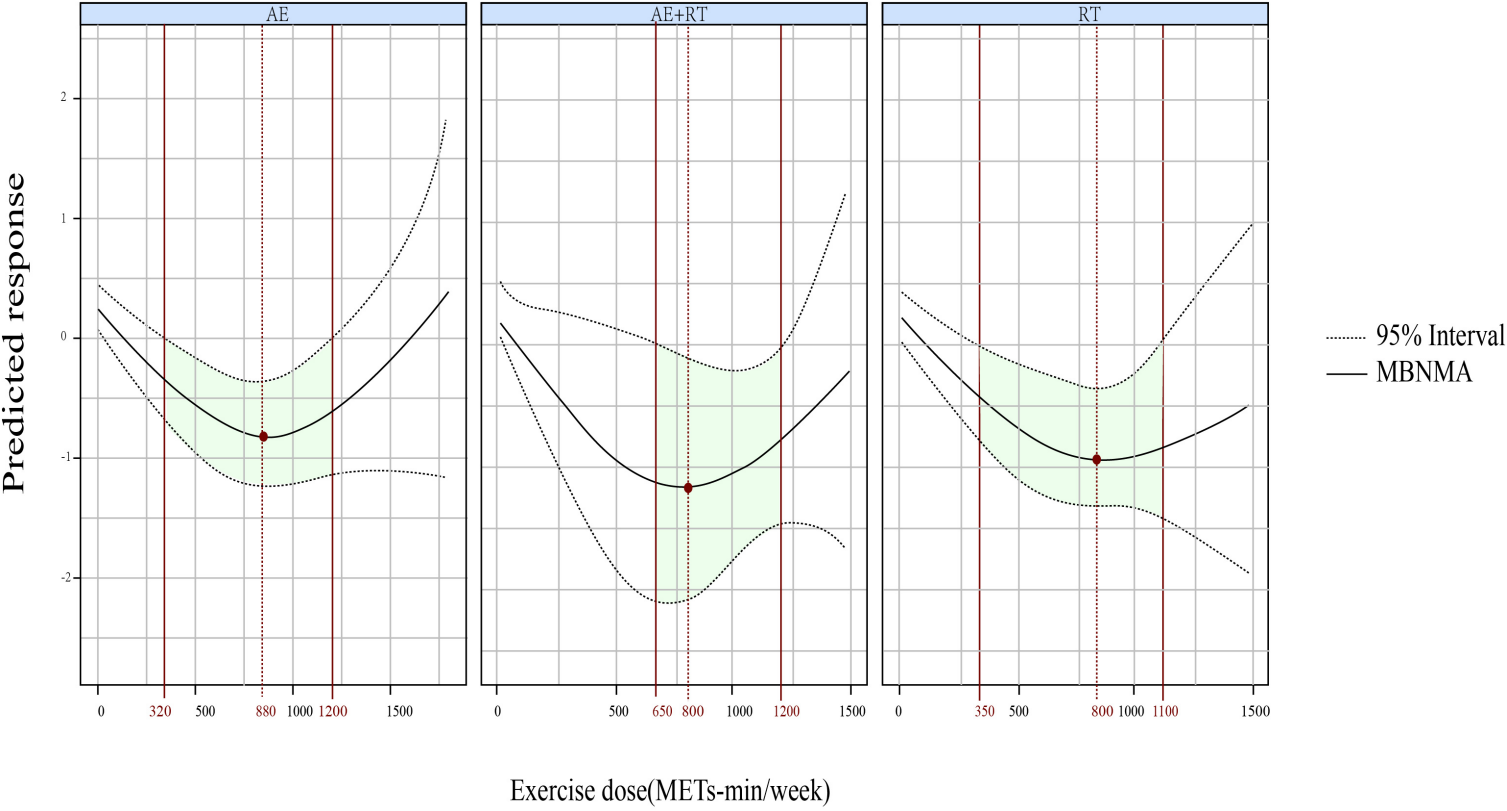
The DR effects of AE, RT, and AE+RT on FBG in individuals with prediabetes. Green-shaded areas indicate significant effectiveness, and red points mark the optimal doses. DR, dose–response; AE, aerobic exercise; RT, resistance training; AE+RT, combined aerobic–resistance training; FBG, fasting blood glucose.

Furthermore, a U-shaped DR curve was identified linking the exercise types AE, AE+RT, and RT to FBG reductions (**Figure 3**). The most significant impact for AE occurred at 880 METs-min/week (SMD, −0.792; 95% CrIs [−1.229 to −0.335]), spanning an effective dose range from 320 to 1,200 METs-min/week. For AE+RT, peak efficacy was recorded at 800 METs-min/week (SMD, −1.097; 95% CrIs [−2.064 to −0.121]), within a beneficial dose window of 650 to 1,200 METs-min/week. The optimal dose for RT was identified as 880 METs-min/week (SMD, −0.910; 95% CrIs [−1.414 to −0.374]), effective from 350 to 1,100 METs-min/week.

#### 2hPG

**Supplementary Figure 4.1.3** presents the direct comparisons among the exercise types and the relevant sample size distributions. The forest and league plots revealed that AE (SMD, −0.71; 95% CrIs [−0.97 to −0.45]), AE+RT (SMD, −0.67; 95% CrIs [−1.17 to −0.17]), and RT (SMD, −0.38; 95% CrIs [−0.69 to −0.07]) all significantly reduced 2hPG versus the Con, with AE achieving the highest ranking (SUCRA, 0.84) (**Supplementary Figure 4.2.1**, **Supplementary Table 4.1.4**).

**Figure 4:**
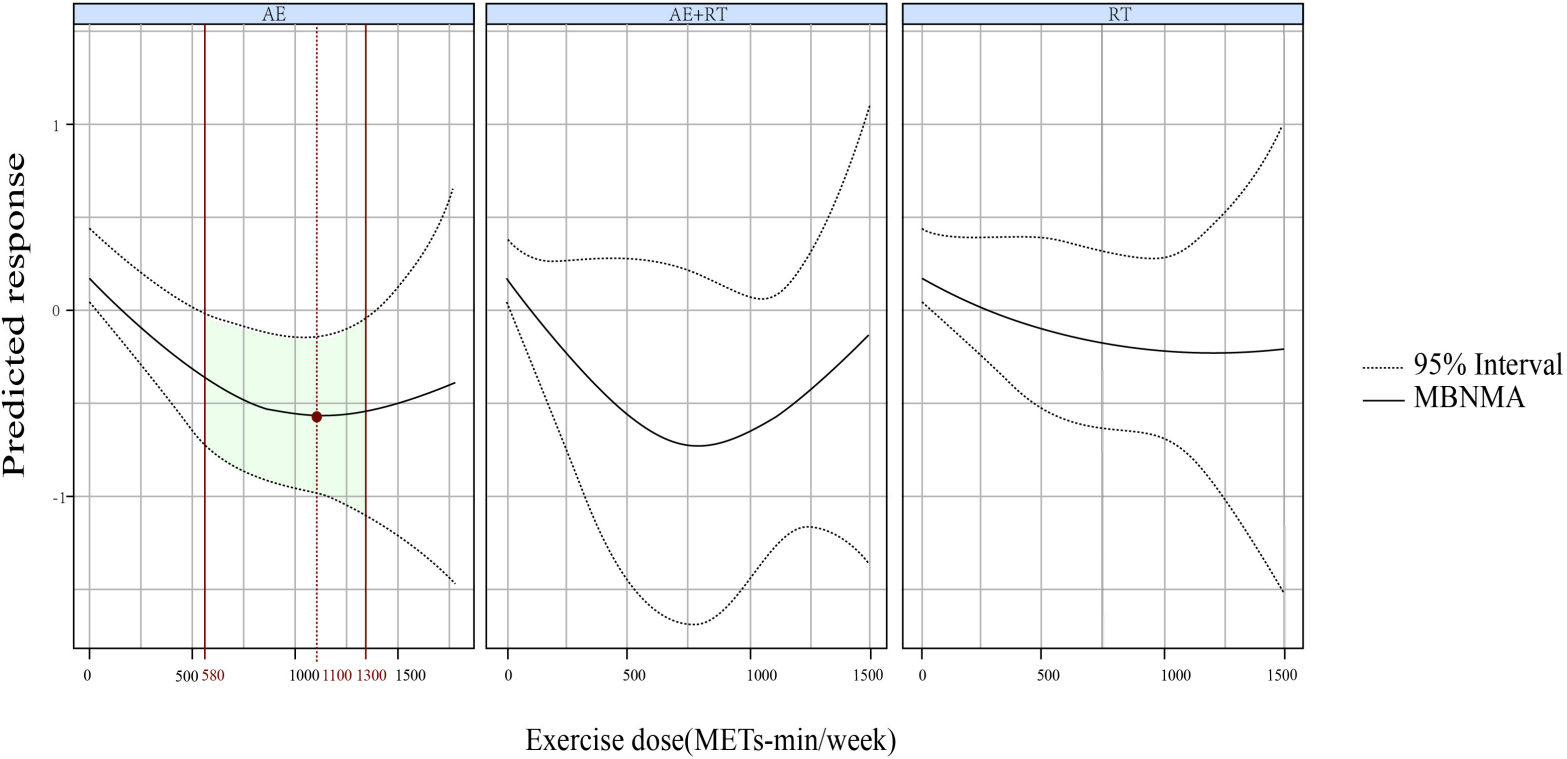
The DR impact of AE, RT, and AE+RT on 2hPG in individuals with prediabetes. Effectiveness is highlighted by green areas, with optimal doses shown in red. DR, dose–response; AE, aerobic exercise; RT, resistance training; AE+RT, combined aerobic–resistance training; 2hPG, 2-hour post-meal blood glucose.

A U-shaped DR curve was also noted between exercise dose and 2hPG for AE (**Figure 4**). The optimal dose was 1,100 METs-min/week (SMD, −0.583; 95% CrIs [−0.986 to −0.185]), with a significant lowering of 2hPG observed within a dose range of 580-1300 METs-min/week. However, neither AE+RT nor RT demonstrated effectiveness in reducing 2hPG across the evaluated range of exercise doses.

#### HbA1c

**Supplementary Figure 4.1.5** presents the direct comparisons and sample size distributions for the various exercise modalities regarding their effects on HbA1c. AE (SMD, −0.30; 95% CrIs [−0.37 to −0.22]), RT (SMD, −0.27; 95% CrIs [−0.46 to −0.09]), and AE+RT (SMD, −0.27; 95% CrIs [−0.36 to −0.17]) significantly outperformed the Con in reducing HbA1c levels, with AE demonstrating the most substantial effect (SUCRA, 0.78) (**Supplementary Figure 4.2.3**, **Supplementary Table 4.1.6**).

**Figure 5:**
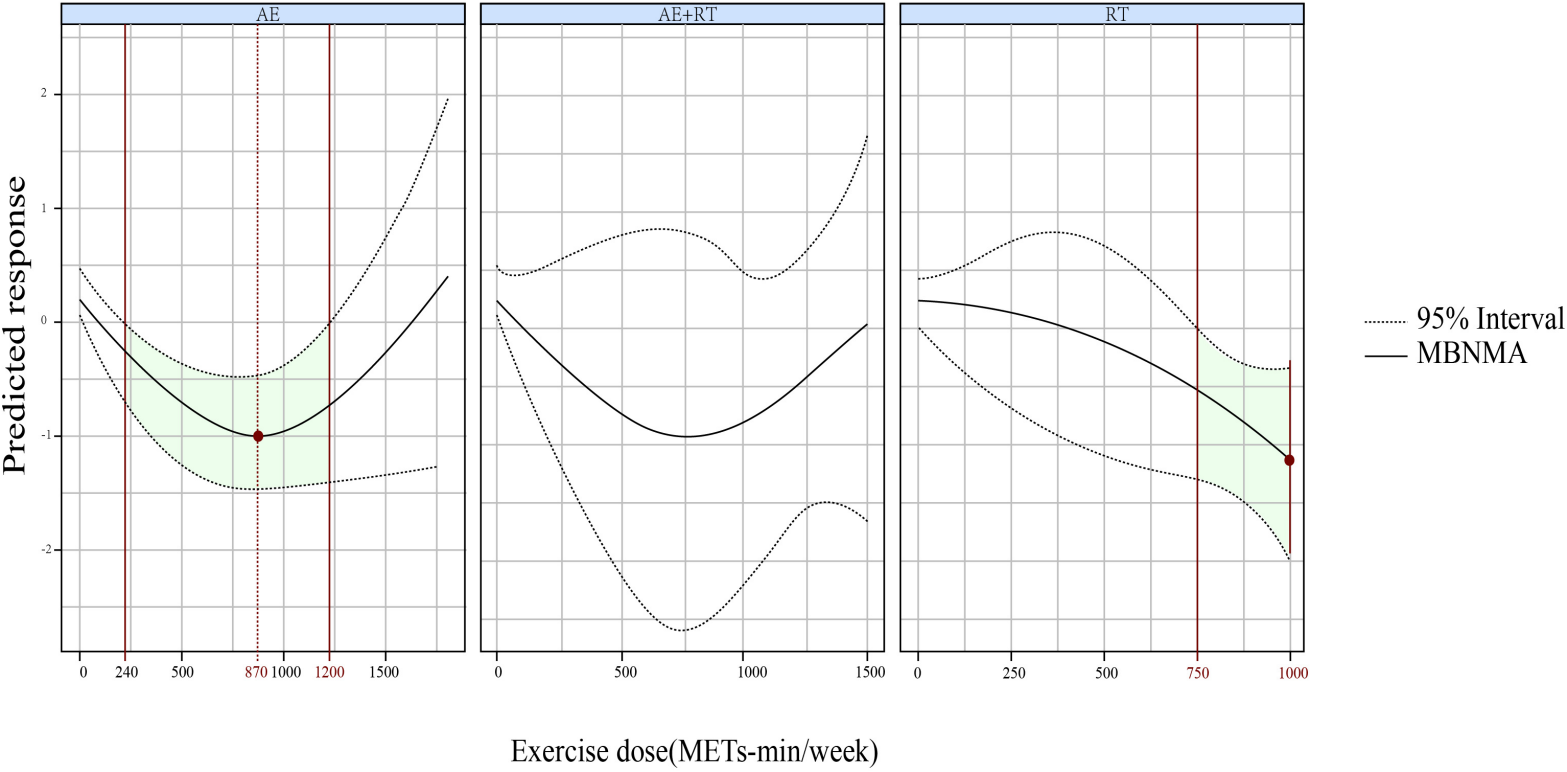
The DRR of AE, RT, and AE+RT on HbA1c levels in individuals with prediabetes. Significant effects are indicated by green shading, and optimal doses are marked in red. DRR, dose–response relationship; AE, aerobic exercise; RT, resistance training; AE+RT, combined aerobic–resistance training; HbA1c, glycosylated hemoglobin A1c.

A U-shaped DR curve also emerged between exercise dose and HbA1c levels for AE (**Figure 5**), where the most robust response occurred at 870 METs-min/week (SMD, −1.015; 95% CrIs [−1.485 to −0.519]), covering an effective dose range of 240–1,200 METs-min/week. Conversely, RT exhibited a non-linear relationship with HbA1c, showing increasing benefits as the exercise dose began at 750 METs-min/week. However, AE+RT did not effectively reduce HbA1c across the assessed exercise dose range.

### Meta-regression and subgroup analysis

The outcomes presented an obvious heterogeneity, including FBG (I^2^ = 85.3%, τ^2^ = 0.4008, p < 0.0001), 2hPG (I^2^ = 81.3%, τ^2^ = 0.1747, p < 0.0001), and HbA1c (I^2^ = 83.9%, τ^2^ = 0.0144, p < 0.0001) (**Supplementary 5**). To determine what variables were responsible for the observed variation in these results, a thorough meta-regression analysis was run. Publication year, average age, male participant %, sample size, exercise program duration, session frequency, and session length were all variables included. This study revealed that the effectiveness of lowering fasting glucose levels was significantly impacted by the frequency of exercise; hence, it was decided to do a subgroup analysis based on exercise frequency. After adjustments for sources of heterogeneity and centering values to the median, the robustness of the results was confirmed, as the conclusions remained consistent. Comparisons across the three metrics showed no evidence of inconsistency (**Supplementary File 7**). Furthermore, comparison-adjusted funnel plots for the three outcomes demonstrated good symmetry, and Egger’s tests for FBG (p = 0.139), 2hPG (p = 0.586), and HbA1c (p = 0.167) indicated no presence of small-study effects (**Supplementary 8**).

## Discussion

### Summary of evidence

This is the inaugural comprehensive DR meta-analysis examining the association between exercise modalities and glycemic regulation for individuals with prediabetes. Our findings demonstrate that AE, RT, and AE+RT significantly improve glucose regulation compared to control. Specifically, AE+RT was most effective in reducing FBG, while AE showed superior efficacy in lowering 2hPG and HbA1c.

We identified a U-shaped DRR for AE+RT, RT, and AE in relation to FBG. AE+RT was most effective at 800 METs-min/week (optimal range, 650–1,200 METs-min/week), while both AE and RT peaked at 880 METs-min/week (effective ranges, 320–1,200 and 350-1,100 METs-min/week, respectively).

For 2hPG, AE exhibited a U-shaped DR curve, with significant reductions observed between 580 and 1,300 METs-min/week, peaking at 1,100 METs-min/week. For HbA1c, RT demonstrated a non-linear decreasing DR pattern, becoming effective at 750 METs-min/week, while AE showed a U-shaped DR, effective between 240 and 1,200 METs-min/week, with maximal efficacy at 870 METs-min/week.

All these will assist in designing personalized exercise prescriptions for glycemic control optimization in people with prediabetes. And based on the principle of exercise prescription and our results, we made some exercise recommendations for the people of prediabetes (**Table 2**).

**Table 2:** Exercise recommendations for improving FBG, 2hPG, and HbA1c in individuals with prediabetes.

Types of prediabetes	Type of physical activity	METs-min/week		Intensity	Energy expenditure (METs-min)	Recommended accumulation (min/week)		Recommendations for exercise prescription (session × mins/per week)	
		Range	Optimal			Minimum	Optimal	Minimum	Optimal
FBG	Aerobic exercise	320–1,200	880	Moderate	4.5 (code 12027,17088)	~70	195	3×~25 5×~15	3×65 5×~40
				Vigorous	8 (code 12025,17028)	~40	110	3×~15 5×~10	3×~35 5×~20
	Resistance training	350–1,100	880	Moderate	5 (code 02052)	~70	176	3×~25 5×~15	3×~60 5×~35
				Vigorous	6 (code 20050)	~30	123	3×10 5×~5	3×~40 5×~25
	Aerobic exercise combined with resistance training	650–1,200	800	Moderate	5	~130	160	3×~45 5×~25	3×~55 5×~30
				Vigorous	8	~80	100	3×~25 5×~15	3×~35 5×20
2hPG	Aerobic exercise	580–1,300	1,100	Moderate	4.5 (code 12027,17088)	~120	244	3×40 5×~25	3×~80 5×~50
				Vigorous	8 (code 12025,17028)	~75	138	3×25 5×15	3×~45 5×~25
HbA1c	Aerobic exercise	240–1,200	870	Moderate	4.5 (code 12027,17088)	~55	193	3×~20 5×~10	3×~65 5×~40
				Vigorous	8 (code 12025,17028)	~30	109	3×10 5×~5	3×~35 5×~20
	Resistance training	750–	NA	Moderate	5 (code 02052)	~150	NA	3×50 5×30	NA
				Vigorous	6 (code 20050)	~125	NA	3×~40 5×25	NA

Intensity coding was extracted from the Compendium of Physical Activity Ainsworth et al. (21). Minutes of the main exercise modalities without considering warm-up and cool-down.

FBG, fasting blood glucose; 2hPG, 2-hour post-meal blood glucose; HbA1c, glycosylated hemoglobin A1c.

### Comparisons with previous studies

Regular exercise can enhance glycemic control, effectively preventing or delaying the onset of T2D (6–10, 33). Our findings support this evidence, identifying AE, RT, and AE+RT as effective interventions, ranked in descending order of efficacy.

AE+RT could the most effectively improve FBG, consistent with prior clinical trials demonstrating its superiority over AE or RT alone (34, 35). AE enhances glucose uptake by increasing insulin receptor numbers and improving insulin sensitivity, while RT builds muscle mass, reducing insulin resistance and promoting glucose transporter type 4 (GLUT4) translocation to muscle cell membranes, thereby enhancing glucose absorption (36–40). The complementary mechanisms of AE and RT likely explain the superior outcomes of combined interventions (41). However, AE+RT demands more time and energy, warranting further investigation into its DRR with FBG in prediabetes.

Notably, our results showed no significant difference in FBG control between AE, RT, and AE+RT at optimal and minimal doses, suggesting that exercise dose may outweigh modality. The optimal AE dose for FBG reduction was 880 METs-min/week, equivalent to ~195 minutes of moderate-intensity or ~110 minutes of vigorous-intensity AE combined with RT weekly. This exceeds previous recommendations for glycemic control (42). Specific to individuals developing impaired fasting glucose (IFG), we recommend ~195 minutes of AE or ~160 minutes of AE+RT weekly. While the American College of Sports Medicine (ACSM) and the American Heart Association (AHA) emphasize the benefits of regular physical activity, middle-aged or older individuals with impaired glucose regulation (IGR) should begin with lower intensities and gradually increase (43).

AE was most effective for improving 2hPG, aligning with studies showing its ability to reduce 2hPG in diabetes and prediabetes (44). This is attributed to enhanced insulin-dependent glucose uptake during meals, driven by increased insulin receptors and sensitivity (45, 46). Our DRR analysis revealed that AE achieves optimal 2hPG reduction at 1,100 METs-min/week (effective range, 590–1,300 METs-min/week). Based on this, we recommend 50 minutes of moderate-intensity walking five times weekly as a practical and achievable strategy.

Finally, AE, AE+RT, and RT all significantly reduced HbA1c levels, reinforcing the benefits of exercise over inactivity for prediabetes management. As HbA1c is the gold standard for blood sugar control, these findings highlight the value of all three modalities. However, given the distinct physiological responses to IFG and impaired glucose tolerance (IGT), we separately analyzed DRRs for FBG and 2hPG. While AE, RT, and AE+RT showed moderate improvements in FBG, 2hPG, and HbA1c, clinicians should interpret these findings cautiously.

### Strengths and limitations

Several strengths in the study enrich the existing research. First, it includes a large sample size, ensuring sufficient statistical power to achieve the research objectives. Second, we employed advanced Bayesian-based DR meta-analysis models, improving the precision of estimates for the relationship between exercise intensity and glycemic control. This approach identified optimal and minimal exercise doses for FBG, 2hPG, and HbA1c, offering practical insights for tailored exercise prescriptions. Third, by incorporating diverse modalities like AE and RT, the study provides flexibility for individuals to choose regimens aligned with their preferences and health needs.

However, some limitations should be noted. First, the reliability of the evidence may be affected by high heterogeneity. Although meta-regression and subgroup analyses focused on elucidating potential sources (such as differences in participant characteristics and exercise protocols), the heterogeneity remains unresolved, highlighting the need for future studies to adopt more comprehensive experimental designs and minimize heterogeneity at the study level. Second, due to the varied combinations of training parameters reported in the included studies, our analysis did not assess interactions between these parameters. It remains unclear whether applying the optimal values for each parameter, as identified in the DRRs, would yield the best outcomes. This underscores the need for future research to develop more advanced analytical techniques to explore these interactions.

In summary, this systematic review together with meta-analysis delineates the DRRs between different exercise modalities (AE, RT, and AE+RT) and key glycemic indicators (FBG, 2hPG, and HbA1c) in individuals with prediabetes, providing a foundation for optimizing exercise prescriptions in this at-risk population.
