# Supplementary material for: Optimal dosage and modality of exercise on glycemic control in people with prediabetes: a systematic review and network meta-analysis
Source: Front Endocrinol (Lausanne). 2025 Apr 28;16:1560676. doi: 10.3389/fendo.2025.1560676 (PMC12066256; doi:10.3389/fendo.2025.1560676)
Supplement: Supplementary file 1 [file DataSheet1.docx]

**Supplementary**

**Table of Contents:**

**[Supplementary 1](#_Toc31822)**

**[Supplementary 1: Search Strategy 1](#_Toc26238)**

*[Database: PubMed <inception to July 15 2024> 1](#_Toc15603)*

*[Database: Ovid MEDLINE(R) <1946 to July 15 2024> 3](#_Toc22073)*

*[Database: Sport Discus <inception to July 15 2024> 4](#_Toc29724)*

*[Cochrane 6](#_Toc13239)*

*[Database: Web of Science <1965 to July 15 2024> 7](#_Toc4179)*

**[Supplementary 2: Characteristics of studies and subjects included in the review 9](#_Toc24616)**

**[Supplementary 3: Risk of Bias 18](#_Toc22496)**

*[The risk of bias assessment for the individual included studies 18](#_Toc12929)*

**[Supplementary 4:Results of direct, indirect, network meta-analysis 23](#_Toc14063)**

**[Supplementary 5: Evaluation of heterogeneity 32](#_Toc973)**

**[Supplementary 6: Network Meta-Regression--changes in heterogeneity 33](#_Toc4882)**

*[Outcome of Network Meta-Regression 33](#_Toc21814)*

**[Supplementary 7: Evaluation of inconsistency...................................................................................46](#_Toc15841)**

**[Supplementary 8: Publication bias](#_Toc10918)** [47](#_Toc10918)

**[Supplementary 9: Grading the evidence for depression symptoms of 50](#_Toc4042)**

**[Supplementary 10: Assessment of Connectivity, Consistency and Transitivity in Network Meta Dose-Response Analysis 55](#_Toc31148)**

**[Supplementary 11: Non-linear functions and models fit comparison 68](#_Toc23781)**

**Supplementary 1: Search Strategy**

## Database: PubMed <inception to July 15 2024>

***Search Strategy:***

| Search number | Query |
| --- | --- |
| 21 | ((Prediabetic State [MeSH Terms]) AND ((((((((((exercise* [MeSH Terms]) OR (resistance training [MeSH Terms])) OR (High-Intensity Interval Training [MeSH Terms])) OR (Circuit-Based Exercise [MeSH Terms])) OR (Tai ji [MeSH Terms])) OR ("aerobic exercise" or "aerobic training" or "Flexibility training" or "multicomponent exercise program" or "multidisciplinary exercise program" or "Nordic Walking" or "Physiotherapy" or "pilates" or "power training" or "treadmill training" or "walking" or "running"))) AND ((((((((randomized controlled trial [Publication Type]) OR (controlled clinical trial[Publication Type])) OR (randomized [Title/Abstract])) OR (placebo [Title/Abstract])) OR (randomly [Title/Abstract])) OR (trial [Title])) OR (clinical trials as topic [MeSH Terms])) NOT ((animals [MeSH Terms]) NOT (humans [MeSH Terms]))) |
| 20 | (((((((randomized controlled trial [Publication Type]) OR (controlled clinical trial [Publication Type])) OR (randomized [Title/Abstract])) OR (placebo [Title/Abstract])) OR (randomly [Title/Abstract])) OR (trial [Title])) OR (clinical trials as topic [MeSH Terms])) NOT ((animals [MeSH Terms]) NOT (humans [MeSH Terms])) |
| 19 | ((((((randomized controlled trial [Publication Type]) OR (controlled clinical trial [Publication Type])) OR (randomized [Title/Abstract])) OR (placebo [Title/Abstract])) OR (randomly [Title/Abstract])) OR (trial [Title])) OR (clinical trials as topic [MeSH Terms]) |
| 18 | ((((((((((exercise*[MeSH Terms]) OR (resistance training [MeSH Terms])) OR (High-Intensity Interval Training [MeSH Terms])) OR (Circuit-Based Exercise [MeSH Terms])) OR (Tai ji [MeSH Terms]))OR ("aerobic exercise" or "aerobic training" or "Flexibility training" or "multicomponent exercise program" or "multidisciplinary exercise program" or "Nordic Walking" or "Physiotherapy" or "pilates" or "power training" or "treadmill training" or "walking" or "running" ) |
| 17 | (animals [MeSH Terms]) NOT (humans [MeSH Terms]) |
| 16 | humans [MeSH Terms] |
| 15 | animals [MeSH Terms] |
| 14 | clinical trials as topic [MeSH Terms] |
| 13 | trial [Title] |
| 12 | randomly [Title/Abstract] |
| 11 | placebo [Title/Abstract] |
| 10 | randomized [Title/Abstract] |
| 9 | controlled clinical trial [Publication Type] |
| 8 | randomized controlled trial [Publication Type] |
| 7 | "aerobic exercise" or "aerobic training" or "Flexibility training" or "multicomponent exercise program" or "multidisciplinary exercise program" or "Nordic Walking" or "Physiotherapy" or "pilates" or "power training" or "treadmill training" or "walking" or "running" |
| 6 | Tai ji [MeSH Terms] |
| 5 | Circuit-Based Exercise [MeSH Terms] |
| 4 | High-Intensity Interval Training [MeSH Terms] |
| 3 | resistance training [MeSH Terms] |
| 2 | exercise* [MeSH Terms] |
| 1 | Prediabetic State [MeSH Terms] |

## Database: Ovid MEDLINE(R) <1946 to July 15 2024>

***Search Strategy:***

1 Prediabetic state.mp.

2 exp Prediabetic state /

3 (aerobic exercise or aerobic training or Flexibility training or exercise$ or resistance training or High-Intensity Interval Training or Circuit-Based Exercise or "multidisciplinary exercise program" or "Nordic Walking" or "Physiotherapy" or "pilates" or "power training" or "treadmill training" or "walking" or "running").mp.

4 exp resistance training/

5 exp exercise$/

6 exp High-Intensity Interval Training /

7 exp Circuit-Based Exercise /

8 exp Tai ji /

9 randomized.ab.

10 randomly.ab.

11 trial.ti.

12 exp clinical trial/

13 exp randomized controlled trials/

14 exp cross-over studies/

15 (clinic$ adj2 trial).mp.

16 (random$ adj5 control$ adj5 trial$).mp.

17 (crossover or cross-over).mp.

18 randomi$.mp.

19 (random$ adj5 (assign$ or allocat$ or assort$ or reciev$)).mp.

20 1 or 2

21 3 or 4 or 5 or 6 or 7 or 8

22 9 or 10 or 11or 12 or 13 or 14 or or 16 or 17 or 18 or 19

23 20 and 21 and 22

## Database: Sport Discus <inception to July 15 2024>

***Search Strategy:***

| S1 | TX "Prediabetic State" | Search modes- Boolean/Phrase |
| --- | --- | --- |
| S2 | TX "exercise*" | Search modes- Boolean/Phrase |
| S3 | TX "aerobic exercise" or "aerobic training" or "Flexibility training" or "multidisciplinary exercise program" or "Nordic Walking" or "Physiotherapy" or "pilates" or "power training" or "treadmill training" or "walking" or "running" | Search modes- Boolean/Phrase |
| S4 | TX "resistance training" | Search modes- Boolean/Phrase |
| S5 | TX "High-Intensity Interval Training" | Search modes- Boolean/Phrase |
| S6 | TX "Circuit-Based Exercise" | Search modes- Boolean/Phrase |
| S7 | TX "physical active" | Search modes- Boolean/Phrase |
| S8 | TX "Tai ji" | Search modes- Boolean/Phrase |
| S9 | S2 OR S3 OR S4 OR S5 OR S6 OR S7 OR S8 | Search modes- Boolean/Phrase |
| S10 | AB "randomized" | Search modes- Boolean/Phrase |
| S11 | AB "randomly" | Search modes- Boolean/Phrase |
| S12 | TI "trial" | Search modes- Boolean/Phrase |
| S13 | AB "clinical trial" | Search modes- Boolean/Phrase |
| S14 | AB "randomized controlled trials" | Search modes- Boolean/Phrase |
| S15 | AB "cross-over studies" | Search modes- Boolean/Phrase |
| S16 | AB "randomi* " | Search modes- Boolean/Phrase |
| S17 | S10 OR S11 OR S12 OR S13 OR S14 OR S15 OR S16 | Search modes- Boolean/Phrase |
| S18 | SU "animals " | Search modes- Boolean/Phrase |
| S19 | S1 AND S9 | Search modes- Boolean/Phrase |
| S20 | S17 AND S19 | Search modes- Boolean/Phrase |
| S22 | S20 NOT S18 | Search modes- Boolean/Phrase |

## Cochrane

#1 MeSH descriptor: [Prediabetic State] explode all trees

#2 ("aerobic exercise" or "aerobic training" or "Flexibility training" or "multidisciplinary exercise program" or "Nordic Walking" or "Physiotherapy" or "pilates" or "power training" or "treadmill training" or "walking" or "running") in Trials (Word variations have been searched)

#3 MeSH descriptor: [resistance training] explode all trees

#4 MeSH descriptor: [exercise] explode all trees

#5 MeSH descriptor: [High-Intensity Interval Training] explode all trees

#6 MeSH descriptor: [Circuit-Based Exercise] explode all trees

#7 MeSH descriptor: [Tai ji] explode all trees

#8 #2 or #3 or #4 or #5 or #6 or #7

#9 #1 and #8

## Database: Web of Science <1965 to July 15 2024>

| # 10 | #9 AND #8 AND #1  Indexes=SCI-EXPANDED, SSCI, A&HCI, CPCI-S, CPCI-SSH, BKCI-S, BKCI-SSH, ESCI, CCR-EXPANDED, IC Timespan=All years |  |  |
| --- | --- | --- | --- |
| # 9 | #7 OR #6 OR #5 OR #4 OR #3 OR #2  Indexes=SCI-EXPANDED, SSCI, A&HCI, CPCI-S, CPCI-SSH, BKCI-S, BKCI-SSH, ESCI, CCR-EXPANDED, IC Timespan=All years |  |  |
| # 8 | TOPIC: (("randomized controlled trial*" or "controlled clinical trial" or "random*" or "clinical trial*" or "randomly" or "trial" or "clinical trial" or "randomized controlled trial*" or "cross-over studies" or "clinic*"))  Indexes=SCI-EXPANDED, SSCI, A&HCI, CPCI-S, CPCI-SSH, BKCI-S, BKCI-SSH, ESCI, CCR-EXPANDED, IC Timespan=All years |  |  |
| # 7 | TOPIC: ("Circuit Based Exercise" or "Circuit-Based Exercises" or "Exercise, Circuit-Based" or "Exercises, Circuit-Based" or "Circuit Training" or "Training, Circuit")  Indexes=SCI-EXPANDED, SSCI, A&HCI, CPCI-S, CPCI-SSH, BKCI-S, BKCI-SSH, ESCI, CCR-EXPANDED, IC Timespan=All years |  |  |
| # 6 | TOPIC: ("High Intensity Interval Training" or "High-Intensity Interval Trainings" or "Interval Training, High-Intensity" or "Training, High-Intensity Interval" or "Trainings, High-Intensity Interval" or "High-Intensity Intermittent Exercise" or "Exercise, High-Intensity Intermittent" or "Exercises, High-Intensity Intermittent" or "High-Intensity Intermittent Exercises" or "Sprint Interval Training" or "Sprint Interval Trainings")  Indexes=SCI-EXPANDED, SSCI, A&HCI, CPCI-S, CPCI-SSH, BKCI-S, BKCI-SSH, ESCI, CCR-EXPANDED, IC Timespan=All years |  |  |
| # 5 | TOPIC: ("Exercise*" or "Exercise Program, Weight-Bearing" or "Exercise Programs, Weight-Bearing" or "Weight Bearing Exercise Program” or "Weight-Bearing Exercise Programs" or "Physical Activity" or "Activities, Physical" or "Activity, Physical" or "Physical Activities" or "Exercise, Physical" or "Exercises, Physical" or "Physical Exercise" or "Physical Exercises" or "Exercise, Isometric" or "Exercises, Isometric" or "Isometric Exercises" or "Isometric Exercise" or "Exercise, Aerobic" or "Aerobic Exercise" or "Aerobic Exercises" or "Exercises, Aerobic" or "Exercise Training" or "Exercise Trainings" or "Training, Exercise" or "Trainings, Exercise")  Indexes=SCI-EXPANDED, SSCI, A&HCI, CPCI-S, CPCI-SSH, BKCI-S, BKCI-SSH, ESCI, CCR-EXPANDED, IC Timespan=All years |  |  |
| # 4 | TOPIC: ("Resistance training" or "Training, Resistance" or "Strength Training" or "Training, Strength" or "Weight-Lifting Strengthening Program" or "Strengthening Program, Weight-Lifting" or "Strengthening Programs, Weight-Lifting" or "Weight Lifting Strengthening Program" or "Weight Lifting Strengthening Programs" or "Weight-Lifting Exercise Program" or "Exercise Program, Weight-Lifting" or "Exercise Programs, Weight-Lifting" or "Weight Lifting Exercise Program" or "Weight-Lifting Exercise Programs" or "Weight-Bearing Strengthening Program" or "Strengthening Program, Weight-Bearing" or "Strengthening Programs, Weight-Bearing" or "Weight Bearing Strengthening Program" or "Weight-Bearing Strengthening Programs" or "Weight-Bearing Exercise Program")  Indexes=SCI-EXPANDED, SSCI, A&HCI, CPCI-S, CPCI-SSH, BKCI-S, BKCI-SSH, ESCI, CCR-EXPANDED, IC Timespan=All years |  |  |
| # 3 | TOPIC: ("Tai ji" or "Tai Chi" or "Chi, Tai" or "Tai Ji Quan" or "Ji Quan, Tai" or "Quan, Tai Ji" or "Taiji" or "Taijiquan" or "T'ai Chi" or "Tai Chi Chuan" )  Indexes=SCI-EXPANDED, SSCI, A&HCI, CPCI-S, CPCI-SSH, BKCI-S, BKCI-SSH, ESCI, CCR-EXPANDED, IC Timespan=All years |  |  |
| # 2 | TOPIC: ("aerobic exercise" or "aerobic training" or "Flexibility training" or "multidisciplinary exercise program" or "Nordic Walking" or "Physiotherapy" or "pilates" or "power training" or "treadmill training" or "walking" or "running")  Indexes=SCI-EXPANDED, SSCI, A&HCI, CPCI-S, CPCI-SSH, BKCI-S, BKCI-SSH, ESCI, CCR-EXPANDED, IC Timespan=All years |  |  |
| # 1 | TOPIC: ("Prediabetic state" or "Prediabetes" or "States, Prediabetic" or "State, Prediabetic" )  Indexes=SCI-EXPANDED, SSCI, A&HCI, CPCI-S, CPCI-SSH, BKCI-S, BKCI-SSH, ESCI, CCR-EXPANDED, IC Timespan=All years |  |  |

# Supplementary 2: Characteristics of studies and subjects included in the review

| **Author**  **Year** | **country** | **Sample Size(men)** | **Age**  **(Mean ± SD)** | **Exercise Prescription** | **Results** |
| --- | --- | --- | --- | --- | --- |
| Tahereh,et al.  2019^1^ | Iran | AE:136(59/77)  Con:136(51/85) | AE:51.3±11.2  Con:53.6±9.4 | AE:60%~70%VO2max,50min/day,3times/week,16weeks | a |
| Juan,et al.  2019^2^ | China | AE:35(10/25)  RT:35(15/20)  Con:35(15/20) | AE:64.23±5.75  RT:62.06±8.11  Con:60.31±7.56 | AE:60%~70%HRmax,50min/day,3times/week,12weeks  RT:50~60%1RM,50min/day,3times/week,12weeks | a,b,c |
| Xia,et al.  2019^3^ | China | AE:34  RT:31  AE+RT:37  Con:35 | 59 | AE:60%~70%HRmax,50min/day,3times/week,2years  RT:60%~80%1RM,60min/day,3times/week,2years  AE+RT:combined AE with RT,30+30min/day,3times/week,2years | a,b,c |
| Kramer,et al.  2018^4^ | USA | AE:88(30/58)  Con:46(14/32) | AE:62.8±12.1  Con:61.9±11.9 | AE:60%~70%HRmax,50min/day,3times/week,18months | a,c |
| Cris A,et al.  2016^5^ | USA | AE1:40(17/23)  AE2:38(15/23) | AE1:61.4±7.1  AE2:60.4±7.0 | AE1:50%VO2reserve,30min/day,3times/week,6months  AE2:75%VO2reserve,60min/day,3times/week,6months | a,b |
| EvaKarin,et al.  2016^6^ | Finland | AE:18(18)  RT:20(20)  Con:17(17) | AT:56±5.6  RT:54±6.2  Con:54±6.9 | AE:55%~75%HRR,50min/day,3times/week,12weeks  RT:50%～851RM,55min/day,3times/week,12weeks | b,c |
| HuoCheng,et al.  2015^7^ | China | AE:60(33/27)  Con:60(35/25) | AE:42.4±5.8  Con:44.1±6.6 | AE:60%~70%HRmax,30min/day,5times/week,12weeks | a,c |
| Herrzig,et al.  2014^8^ | Finland | AE:33(9/24)  Con:35(9/26) | AE:58.1±9.9  Con:59.5±10.8 | AE:60%~70%HRmax,40~45min/day,3times/week,12weeks | a,b |
| Mika,et al.  2013^9^ | Finland | AE:39(39)  RT:36(36)  Con:40(40) | AE:55±6.2  RT:54±6.1  Con:54±7.2 | AE:65%~75%HRR,50min/day,3times/week,12weeks  RT:75%~85%1RM,50min/day,3times/week,12weeks | a,b,c |
| Fritz,et al.  2013^10^ | Sweden | AE:14(5/9)  Con:21(10/11) | AE:59.1±6.2  Con:61.8±3.4 | AE:60%~70%HRmax,60min/day,5times/week,16weeks | a,b,c |
| Hansen,et al.  2012^11^ | Sweden | RT1:9(2/7)  RT2:9(2/7)  Con:9(2/7) | RT1:59.1±6.2  RT2:61.8±3.4  Con:56.1±4.4 | RT1:85%1RM,60min/day,3times/week,16weeks  RT2:65%1RM,60min/day,3times/week,16weeks | a,b |
| Cristian,et al.  2012^12^ | Chile | AE:12(0/12)  RT:8(0/8)  AE+RT:10(0/10)  Con:13(0/13) | AE:39.2±9.5  RT:33.9±9.3  AE+RT:43.3±8.1  Con:40.1±11.4 | AE:>80%HRmax,20min/day,3times/week,12week  RT:75%~85%1RM,45min/day,2times/week,12week  AE+RT:combined AE with RT,20~45min/day,5times/week,12weeks | a,c |
| Martin,et al.  2009^13^ | Austria | AE+RT:18(8/10)  Con:18(8/10) | AE+RT:55.8±5.5  Con:59.1±7.8 | AE+RT:70%HRmax+70%1RM,60min/day,3times/week,12months | a |
| Desch,et al.  2010^14^ | Germany | AE:14(11/3)  Con:12(8/4) | AE:62.3±6.2  Con:62.3±6.5 | AE:75%VO2max,90min/day,3times/week,6months | a,b,c |
| Eriksson,et al.  1998^15^ | Finland | AE:7(3/4)  RT:7(4/3)  Con:8(8/0) | AE:60±5  RT:40±3  Con:60±5 | AE:60%HRmax,60min/day,3times/week,24weeks  RT:50%~60%1RM,60min/day,3times/week,10weeks | a |
| Steven K,et al.  2012^16^ | USA | AE+RT:8(3/5)  Con:8(2/6) | AE+RT:45.4±8.0  Con:49.8±10.9 | AE+RT:70%HRmax+70%1RM,45+15min/day,3times/week,12weeks | a |
| Robin L,et al.  2009^17^ | USA | RT:10(0/10)  Con:6(0/6) | RT:56.3±6.4  Con:53.2±6.5 | RT:85%1RM,30min/day,3times/week,12weeks | a |
| Roumen,et al.  2008^18^ | Dutch | AE:54(30/24)  Con:52(28/24) | AE:58.4±6.8  Con:54.2±5.8 | AE:70%VO2max,30min/day,5times/week,3years | b,c |
| Rowan,et al.  2017^19^ | Canada | AE1:10  AE2:11 | AE1:47.7±6.93  AE2:53.6±8.21 | AE1:60%~70%HRR,28min/day,3times/week,12weeks  AE2:16min90%HRR+12min50%~60%HRR,28min/day,3times/week,  12weeks | a,b |
| XiaoDan,et al.  2020^20^ | China | AE:83(24/59)  RT:82(30/52)  Con:83(33/50) | AE:60.93±5.71  RT:59.91±5.92  Con:60.73±5.83 | AE:60%~70%HRmax,50min/day,3times/week,6months  RT:50%~60%1RM,50min/day,3times/week,6months | a,b,c |
| sulin,et al.  2017^21^ | China | AE:29(6/23)  Con:29(7/22) | AE:59±4.4  Con:60±3.4 | AE:60%~75%VO2max,30~60min/day,3times/week,6months | a,b,c |
| Lin,et al.  2021^22^ | China | AE:43(3/40)  RT:42(4/38)  Con:43(4/39) | AE:60.35±4.29  RT:60.12±3.97  Con:59.94±4.40 | AE:60%~70%HRmax,50min/day,3times/week,12months  RT:60%~80%1RM,50min/day,3times/week,12months | a,b,c |
| Nicole M,et al.  2019^23^ | USA | AE1:17  AE2:12 | AE1:45.7±4.4  AE2:50.8±4.4 | AE1:79.8±3.3%HRmax,14~41min/day,3times/week,16weeks  AE2:53.1±2.3%HRR,30-60min/day,3times/week,16weeks | a,c |
| XiJuan,et al.  2023^24^ | China | AE:26(15/11)  RT:23(13/10)  Con:21(11/10) | AE:51±9.5  RT:52±8.2  Con:50±6.5 | AE:40%~59%VO2max,50min/day,3times/week,12weeks  RT:50%~60%1RM,50min/day,3times/week,12weeks | a,b |
| YanWei,et al.  2023^25^ | China | AE+RT:17(8/9)  Con:17(5/12) | AE+RT:63.41±5.06  Con:61.82±4.33 | AE+RT:60%~70%HRmax,47~76min/day,5times/week,6months | a,b,c |
| XiaoJun,et al.  2022^26^ | China | AE1:32(14/28)  AE2:34(16/18)  Con:32(11/21) | AE1:59.81±4.54  AE2:59.18±3.93  Con:59.09±5.25 | AE1:60%~70%HRmax,40~45min/day,15times/mouth,12months  AE2:50%~60%HRmax,40-60min/day,15times/mouth,12months | a,c |
| YiPing,et al.  2013^27^ | China | AE:20  AE+RT:20  Con:21 | 49.8±4.8 | AE:60~70%HRmax,50min/day,4times/week,24weeks | a,c |
| Mehdi,et al.  2022^28^ | Iran | AE:22(22/0)  Con:20(20/0) | 45-55 | AE:50~75%HRR,40min/day,3times/week,12weeks | a,c |
| BoRam,et al.  2022^29^ | Korea | AE:15(0/15)  RT:12(0/12)  Con:9(0/9) | AE:70.47±5.57  RT:72.25±5.07  Con:67.78±2.33 | AE:55∼65%HRR;250∼30kcal/day  RT:55∼65%1RM;250∼300kcal/day,40min/day,3times/week,12weeks | c |
| Xue,et al.  2020^30^ | China | AE:83(24/59)  RT:82(30/52)  Con:83(33/50) | AE:60.93±5.71  RT:59.91±5.92  Con:60.73±5.83 | AE:60–70%HRmax  RT:50~60%1RM,50min/day,3times/week,24months | a,b,c |

*AE* Aerobic Exercise, *RT* Resistance Training, *AE+RT* Combined aerobic exercise with resistance training, *Con* control group, *HRR* Reserve heart rate, *HRmax* Maximum heart rate, *1RM* One-repetition maximum, *a* Fasting blood glucose, *b* 2-hour postprandial blood glucose, *c* glycosylated hemoglobin.

# Supplementary 3: Risk of Bias

## Table 3.1 The risk of bias assessment for the individual included studies

| **Study** | **Randomization process** | **Deviations from intended interventions** | **Missing outcome data** | **Measurement of the outcome** | **Selection of the reported result** | **Overall Bias** |
| --- | --- | --- | --- | --- | --- | --- |
| Tahereh,et al.(2019)^1^ | Low | Low | Low | Low | Low | Low |
| Juan,et al.(2019)^2^ | Some concerns | Low | Low | Low | Low | Some concerns |
| Xia,et al.(2019)^3^ | Low | Low | Low | Low | Low | Low |
| Kramer,etal.(2018)^4^ | Some concerns | Low | Low | Low | Low | Some concerns |
| Cris A,et al.(2016)^5^ | Low | Some concerns | Low | Low | Low | Some concerns |
| EvaKarin,etal.(2016)^6^ | Some concerns | Some concerns | Low | Low | Some concerns | Some concerns |
| HuoCheng,et al.(2015)^7^ | Some concerns | Low | Low | Low | Some concerns | Some concerns |
| Herrzig,et al.(2014)^8^ | Some concerns | Low | Low | Low | Some concerns | Some concerns |
| Mika,et al.(2013)^9^ | Some concerns | Low | Low | Low | Some concerns | Some concerns |
| Fritz,et al.(2013)^10^ | Low | Low | Low | Low | Low | Low |
| Elisabeth,et al.(2012)^11^ | Low | Low | Low | Low | Low | Low |
| Cristian,et al.(2012)^12^ | Some concerns | Low | Low | Low | Some concerns | Some concerns |
| Martin,et al.(2009)^13^ | Some concerns | Low | Low | Low | Low | Some concerns |
| Desch,et al.(2010)^14^ | Some concerns | Low | Low | Low | Low | Some concerns |
| Eriksson,et al.(1998)^15^ | Some concerns | Low | Low | Low | Low | Some concerns |
| Steven K,et al.(2012)^16^ | Low | Low | Low | Low | Low | Low |
| Robin L,et al.(2009)^17^ | Some concerns | Low | Low | Low | Low | Some concerns |
| Roumen,et al.(2008)^18^ | Some concerns | Low | Low | Low | Some concerns | Some concerns |
| Rowan,et al.(2017)^19^ | Some concerns | Low | Low | Low | Some concerns | Some concerns |
| XiaoDan,et al.(2020)^20^ | Some concerns | Low | Low | Low | Low | Some concerns |
| SuLin,et al.(2017)^21^ | High | Low | Low | Low | Low | High |
| Lin,et al.(2021)^22^ | Some concerns | Low | Low | Low | Some concerns | Some concerns |
| Nicole M,et al.(2019)^23^ | Some concerns | Low | Low | Low | Some concerns | Some concerns |
| XiJuan,et al.(2023)^24^ | Some concerns | Low | Low | Low | Some concerns | Some concerns |
| XiaoJun,et al.(2022)^25^ | Some concerns | Low | Low | Low | Some concerns | Some concerns |
| Dunne, et al. (2016) ^26^ | Some concerns | Low | Low | Low | Some concerns | Some concerns |
| YiPing,et al.(2013)^27^ | Some concerns | Some concerns | Low | Low | Some concerns | Some concerns |
| Mehdi,et al.(2022)^28^ | Some concerns | Some concerns | Low | Low | Low | Some concerns |
| BoRam,et al.(2022)^29^ | Some concerns | Some concerns | Low | Low | Some concerns | Some concerns |
| Xue,et al.(2020)^30^ | Some concerns | Some concerns | Low | Low | Some concerns | Some concerns |

**List of included studies**

1. Shamizadeh T, Jahangiry L, Sarbakhsh P, Ponnet K: Social cognitive theory-based intervention to promote physical activity among prediabetic rural people: a cluster randomized controlled trial. Trials 2019.<https://doi.org/10.1186/s13063-019-3220-z>

2. Yan J, Dai X, Feng J, Yuan X, Li J, Yang L, Zuo P, Fang Z, Liu C, Hsue C et al: Effect of 12-Month Resistance Training on Changes in Abdominal Adipose Tissue and Metabolic Variables in Patients with Prediabetes: A Randomized Controlled Trial. J Diabetes Res 2019.<https://doi.org/10.1155/2019/8469739>

3. Dai X, Zhai L, Chen Q, Miller JD, Lu L, Hsue C, Liu L, Yuan X, Wei W, Ma X et al: Two-year-supervised resistance training prevented diabetes incidence in people with prediabetes: A randomised control trial. Diabetes Metab Res Rev 2019.<https://doi.org/10.1002/dmrr.3143>

4. Kramer MK, Vanderwood KK, Arena VC, Miller RG, Meehan R, Eaglehouse YL, Schafer G, Venditti EM, Kriska AM: Evaluation of a Diabetes Prevention Program Lifestyle Intervention in Older Adults: A Randomized Controlled Study in Three Senior/Community Centers of Varying Socioeconomic Status. Diabetes Educ 2018.<https://doi.org/10.1177/0145721718759982>

5. Slentz CA, Bateman LA, Willis LH, Granville EO, Piner LW, Samsa GP, Setji TL, Muehlbauer MJ, Huffman KM, Bales CW et al: Effects of exercise training alone vs a combined exercise and nutritional lifestyle intervention on glucose homeostasis in prediabetic individuals: a randomised controlled trial. Diabetologia 2016.<https://doi.org/10.1007/s00125-016-4051-z>

6. Gidlund E-K, von Walden F, Venojärvi M, Risérus U, Heinonen OJ, Norrbom J, Sundberg CJ: Humanin skeletal muscle protein levels increase after resistance training in men with impaired glucose metabolism. Physiol Rep 2016.

7. Chen K-M, Chen M-H, Chao H-C, Hung H-M, Lin H-S, Li C-H. Sleep quality, depression state, and health status of older adults after silver yoga exercises: cluster randomized trial. Int J Nurs Stud. 2009;46(2):154-163. doi: 10.1016/j.ijnurstu.2008.09.005.

8. Herzig KH, Ahola R, Leppäluoto J, Jokelainen J, Jämsä T, Keinänen-Kiukaanniemi S: Light physical activity determined by a motion sensor decreases insulin resistance, improves lipid homeostasis and reduces visceral fat in high-risk subjects: PreDiabEx study RCT. Int J Obes (Lond) 2014.<https://doi.org/10.1038/ijo.2013.224>

9. Venojärvi M, Wasenius N, Manderoos S, Heinonen OJ, Hernelahti M, Lindholm H, Surakka J, Lindström J, Aunola S, Atalay M et al: Nordic walking decreased circulating chemerin and leptin concentrations in middle-aged men with impaired glucose regulation. Ann Med 2013.<https://doi.org/10.3109/07853890.2012.727020>

10. Fritz T, Caidahl K, Krook A, Lundström P, Mashili F, Osler M, Szekeres FLM, Östenson CG, Wändell P, Zierath JR: Effects of Nordic walking on cardiovascular risk factors in overweight individuals with type 2 diabetes, impaired or normal glucose tolerance. Diabetes Metab Res Rev 2013.<https://doi.org/10.1002/dmrr.2321>

11. Hansen E, Landstad BJ, Gundersen KT, Torjesen PA, Svebak S: Insulin sensitivity after maximal and endurance resistance training. J Strength Cond Res 2012.<https://doi.org/10.1519/JSC.0b013e318220e70f>

12. Alvarez C, Ramírez R, Flores M, Zúñiga C, Celis-Morales CA: [Effect of sprint interval training and resistance exercise on metabolic markers in overweight women]. Rev Med Chil 2012.<https://doi.org/10.4067/S0034-98872012001000008>

13. Burtscher M, Gatterer H, Kunczicky H, Brandstätter E, Ulmer H: Supervised exercise in patients with impaired fasting glucose: impact on exercise capacity. Clin J Sport Med 2009.<https://doi.org/10.1097/JSM.0b013e3181b8b6dc>

14. Desch S, Sonnabend M, Niebauer J, Sixt S, Sareban M, Eitel I, de Waha S, Thiele H, Blüher M, Schuler G: Effects of physical exercise versus rosiglitazone on endothelial function in coronary artery disease patients with prediabetes. Diabetes Obes Metab 2010.<https://doi.org/10.1111/j.1463-1326.2010.01234.x>

15. Eriksson J, Tuominen J, Valle T, Sundberg S, Sovijärvi A, Lindholm H, Tuomilehto J, Koivisto V: Aerobic endurance exercise or circuit-type resistance training for individuals with impaired glucose tolerance? Horm Metab Res 1998.

16. Malin SK, Gerber R, Chipkin SR, Braun B: Independent and combined effects of exercise training and metformin on insulin sensitivity in individuals with prediabetes. Diabetes Care 2012.<https://doi.org/10.2337/dc11-0925>

17. Marcus RL, Lastayo PC, Dibble LE, Hill L, McClain DA: Increased strength and physical performance with eccentric training in women with impaired glucose tolerance: a pilot study. J Womens Health (Larchmt) 2009.<https://doi.org/10.1089/jwh.2007.0669>

18. Roumen C, Corpeleijn E, Feskens EJM, Mensink M, Saris WHM, Blaak EE: Impact of 3-year lifestyle intervention on postprandial glucose metabolism: the SLIM study. Diabet Med 2008.<https://doi.org/10.1111/j.1464-5491.2008.02417.x>

19. Rowan CP, Riddell MC, Gledhill N, Jamnik VK: Aerobic Exercise Training Modalities and Prediabetes Risk Reduction. Med Sci Sports Exerc 2017.<https://doi.org/10.1249/MSS.0000000000001135>

20. Yuan X, Dai X, Liu L, Hsue C, Miller JD, Fang Z, Li J, Feng J, Huang Y, Liu C et al: Comparing the effects of 6 months aerobic exercise and resistance training on metabolic control and β-cell function in Chinese patients with prediabetes: A multicenter randomized controlled trial. J Diabetes 2020.<https://doi.org/10.1111/1753-0407.12955>

21. Cheng S, Ge J, Zhao C, Le S, Yang Y, Ke D, Wu N, Tan X, Zhang X, Du X et al: Effect of aerobic exercise and diet on liver fat in pre-diabetic patients with non-alcoholic-fatty-liver-disease: A randomized controlled trial. Scientific Reports 2017.<https://doi.org/10.1038/s41598-017-16159-x>

22. Liu L, Ma X, Xu H, Ruan S, Yuan X: Comparing the effects of 12 months aerobic exercise and resistance training on glucose metabolism among prediabetes phenotype: A explorative randomized controlled trial. Prim Care Diabetes 2021.<https://doi.org/10.1016/j.pcd.2020.11.003>

23. Gilbertson NM, Mandelson JA, Hilovsky K, Akers JD, Hargens TA, Wenos DL, Edwards ES: Combining supervised run interval training or moderate-intensity continuous training with the diabetes prevention program on clinical outcomes. Eur J Appl Physiol 2019.<https://doi.org/10.1007/s00421-019-04137-2>

24.Luo, X., Wang, Z., Li, B. et al. Effect of resistance vs. aerobic exercise in pre-diabetes: an RCT. Trials 24, 110 (2023). https://doi.org/10.1186/s13063-023-07116-3

25.Cai, Y., Wang, S., Wang, S., Gu, Q., Huang, Y., Li, J., Wang, R., & Liu, X. (2023). Effects of yijinjing combined with resistance training on body fat distribution and hepatic lipids in middle-aged and older people with prediabetes mellitus: A randomized controlled trial. Experimental Gerontology, 179, 112250. <https://doi.org/10.1016/j.exger.2023.112250>

26.Ma, X., Li, M., Liu, L., Lei, F., Wang, L., Xiao, W., Tan, Y., He, B., & Ruan, S. (2022). A randomized controlled trial of baduanjin exercise to reduce the risk of atherosclerotic cardiovascular disease in patients with prediabetes. Scientific Reports, 12(1), 19338. <https://doi.org/10.1038/s41598-022-22896-5>

27.Liu, Y., Li, J., Zhang, Z., Tang, Y., Chen, Z., & Wang, Z. (2013). Effects of exercise intervention on vascular endothelium functions of patients with impaired glucose tolerance during prediabetes mellitus. Experimental and Therapeutic Medicine, 5(6), 1559–1565. <https://doi.org/10.3892/etm.2013.1064>

28.Kargarfard, M., Nobari, H., Kamyar, K., Zadeh, A. K., & Oliveira, R. (2022). Effects of 12-week moderate aerobic exercise on ROCK2 activity, hs-CRP and glycemic index in obese patients with impaired glucose tolerance. Physiology and Behavior, 257, 113976. <https://doi.org/10.1016/j.physbeh.2022.113976>

29.Kim, B.-R., & Lim, S.-T. (2022). Effects of leisure-time physical activity on cognitive reserve biomarkers and leisure motivation in the pre-diabetes elderly. Health Care, 10(4), 737. <https://doi.org/10.3390/healthcare10040737>

30.Chen, X., Zhao, S., Hsue, C., Dai, X., Liu, L., Miller, J. D., Fang, Z., Feng, J., Huang, Y., Wang, X., & Lou, Q. (2021). Effects of aerobic training and resistance training in reducing cardiovascular disease risk for patients with prediabetes: A multi-center randomized controlled trial. Primary Care Diabetes, 15(6), 1063–1070. <https://doi.org/10.1016/j.pcd.2021.08.013>

# Supplementary 4:Results of direct,indirect,network meta-analysis

## 4.1 Results of network meta-analysis

## 4.1.1 Network plot of FBG


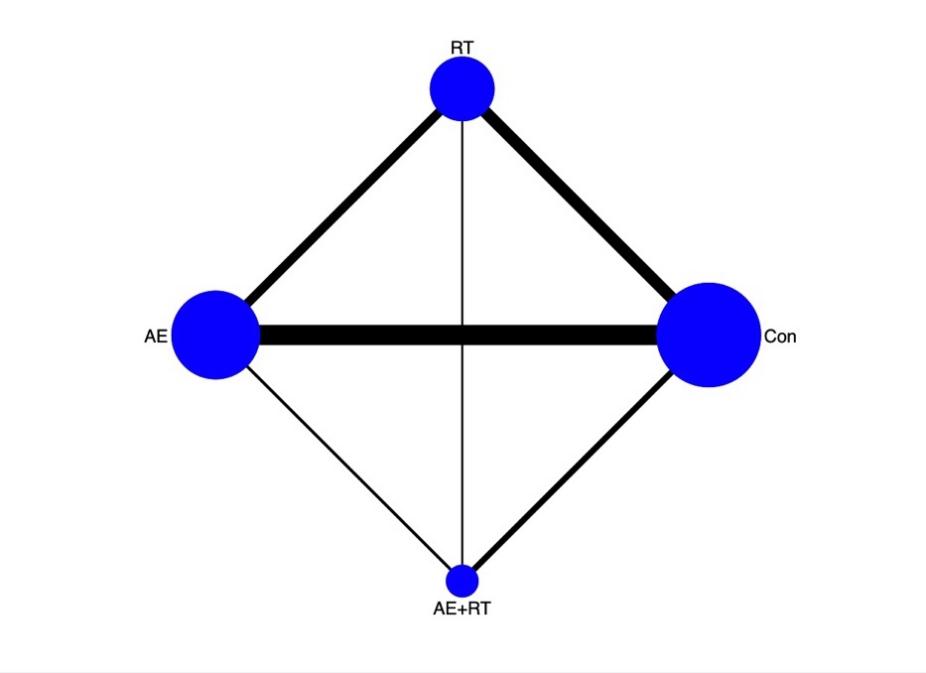


**Figure 4.1.1** Network plot of FBG. The size of the modes corresponds to the number of participants randomized to each physical activity type. Physical activity type with direct comparisons are linked with a line; Its thickness corresponds to the number of trials evaluating the comparison. *AE*: Aerobic exercise, *RT*: Resistance training, *AE+RT*: Aerobic exercise combined with resistance training, *Con*: No exercise.

## 4.1.2 The league table of FBG


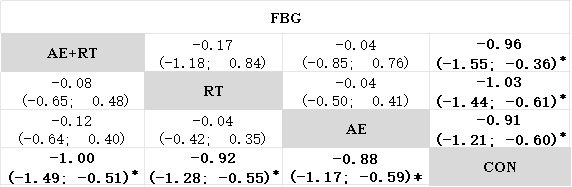


## Table 4.1.2 The league table of FBG

## 4.1.3 Network plot of 2hPG


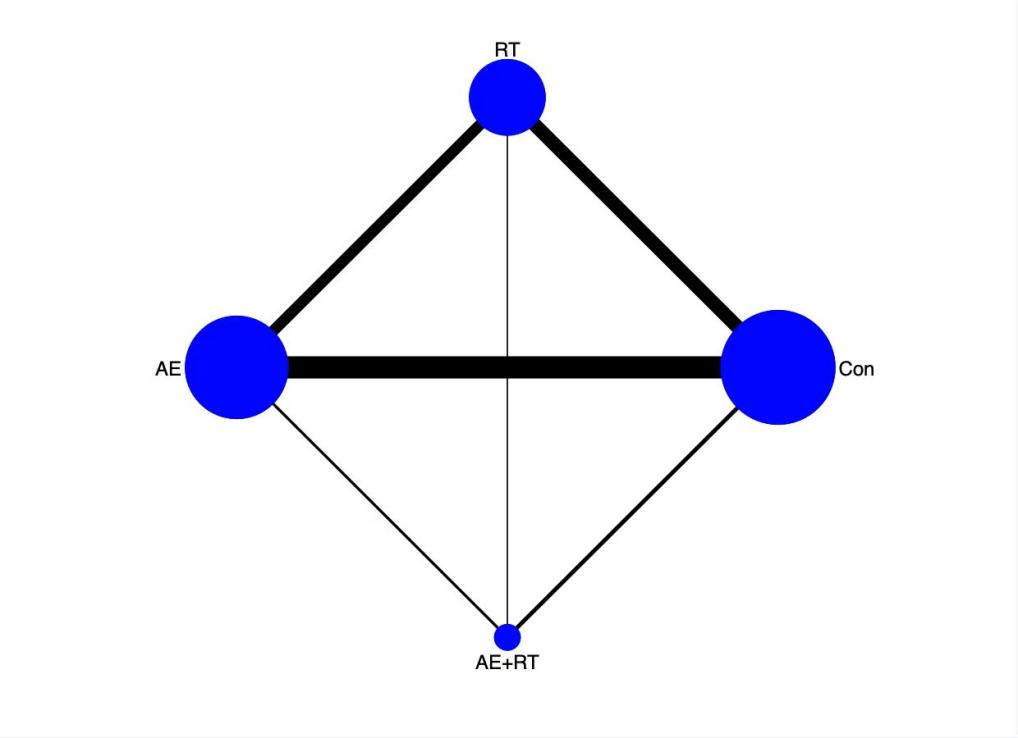


**Figure 4.1.3** Network plot of 2hPG. The size of the modes corresponds to the number of participants randomized to each physical activity type. Physical activity type with direct comparisons are linked with a line; Its thickness corresponds to the number of trials evaluating the comparison. *AE*: Aerobic exercise, *RT*: Resistance training, *AE+RT*: Aerobic exercise combined with resistance training, *Con*: No exercise.

## 4.1.4 The league table of 2hPG

##
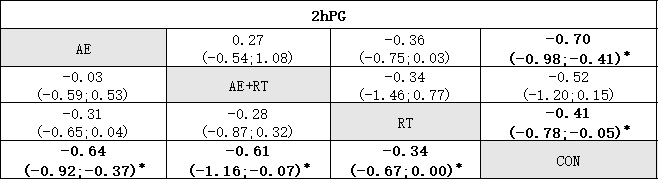


## Table 4.1.4 The league table of 2hPG

## 4.1.5 Network plot of HbA1c


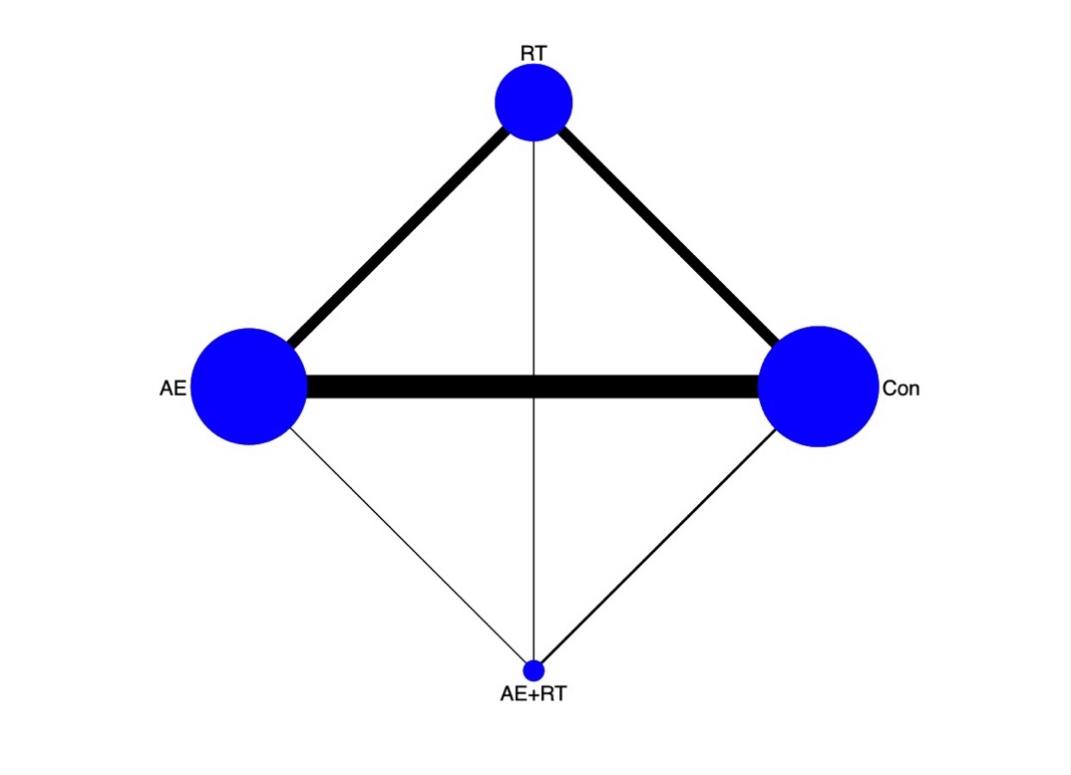


**Figure 4.1.5** Network plot of HbA1c. The size of the modes corresponds to the number of participants randomized to each physical activity type. Physical activity type with direct comparisons are linked with a line; Its thickness corresponds to the number of trials evaluating the comparison. *AE*: Aerobic exercise, *RT*: Resistance training, *AE+RT*: Aerobic exercise combined with resistance training, *Con*: No exercise.

## 4.1.6 The league table of HbA1c


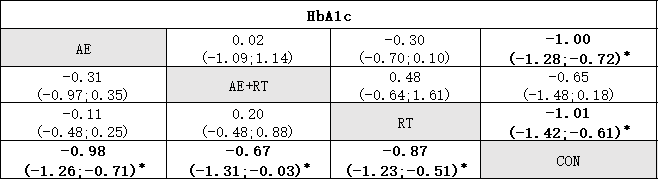


## Table 4.1.6 The league table of HbA1c

## 4.2 Forest plots

# 4.2.1 Forest plot of outcome FBG


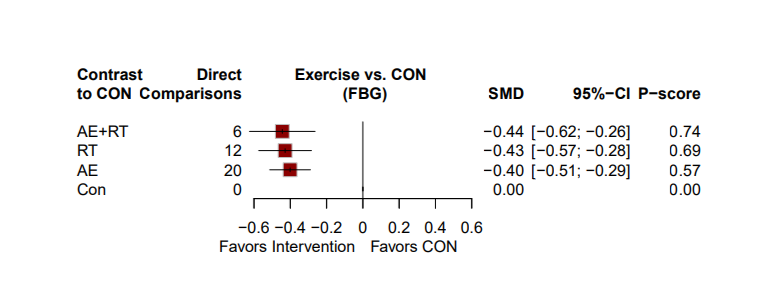


**Figure 4.2.1** Exercise training type are ranked according to SMD compared to CON. Treatments crossing the y-axis are not significantly different from CON. *SMD*: Standardized Mean Difference, *AE*: Aerobic exercise, *RT*: Resistance training, *AE+RT*: Aerobic exercise combined with resistance training, *Con*: No exercise.

# 4.2.2 Forest plot of outcome 2hPG


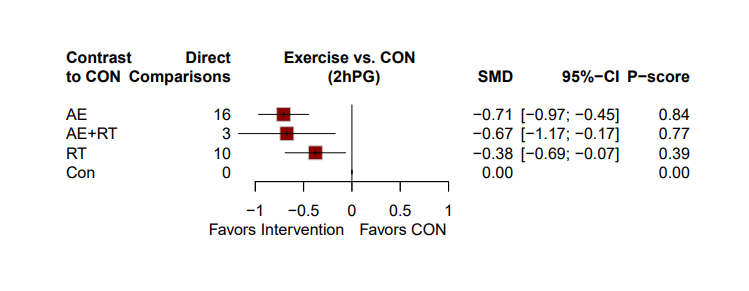


**Figure 4.2.2** Exercise training type are ranked according to SMD compared to CON. Treatments crossing the y-axis are not significantly different from CON. *SMD*: Standardized Mean Difference, *AE*: Aerobic exercise, *RT*: Resistance training, *AE+RT*: Aerobic exercise combined with resistance training, *Con*: No exercise.

**4.2.3 Forest plot of outcome HbA1c**

**
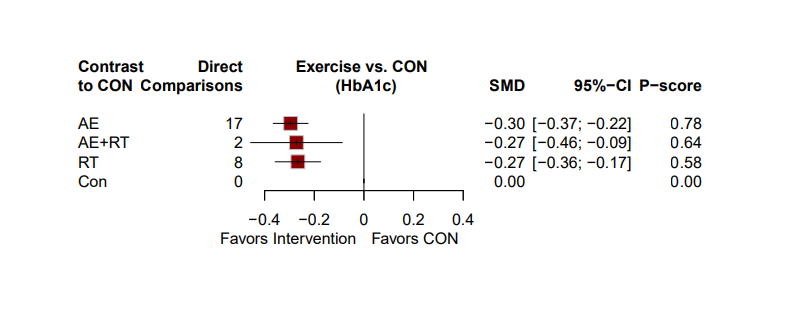
**

**Figure 4.2.3** Exercise training type are ranked according to SMD compared to CON. Treatments crossing the y-axis are not significantly different from CON. *SMD*: Standardized Mean Difference, *AE*: Aerobic exercise, *RT*: Resistance training, *AE+RT*: Aerobic exercise combined with resistance training, *Con*: No exercise.

# Supplementary 5: Evaluation of heterogeneity

We use the tau square (τ^2^) test and p-value to qualitatively analyze the statistical heterogeneity between the studies. The larger the τ^2^ and the smaller the p-value, the greater the possibility of heterogeneity; on the contrary, the smaller the existence heterogeneity. In addition, I^2^ is a parameter for quantitative analysis of the heterogeneity between the results of each study. It’ s value is distributed from 0-100%. When I^2^ is less than 25%, it means that the heterogeneity is low; 25%-50% means that the heterogeneity is moderate; I^2^ > 75% means high heterogeneity. In summary, when I^2^ > 50%, it means that there is substantial heterogeneity.

| Primary outcomes | τ^2^ | Q | df | P | I^2^ | Heterogeneity assessment |
| --- | --- | --- | --- | --- | --- | --- |
| FBG | 0.4008 | 265.86 | 39 | 0.0001 | 85.3% | High |
| 2hPG | 0.1747 | 149.38 | 28 | 0.0001 | 81.3% | High |
| HbA1c | 0.0144 | 161.64 | 26 | 0.0001 | 83.9% | High |

# Supplementary 6: Network Meta-Regression--changes in heterogeneity

# 6.1 Outcome of Network Meta-Regression

| Covariate | Shared beta ( median and 95%Crl ) | | |
| --- | --- | --- | --- |
|  | FBG | 2hPG | HbA1c |
| Publish Year | 0.189(-0.382,0.729) | 0.115(-0.387,0.592) | 0.347(-0.250,0.948) |
| Sampe Size | 0.268(-0.192,0.696) | 0.167(-0.276,0.592) | -0.417(-1.248,0.288) |
| Percentage of Male | 0.368(-0.179,0.919) | -0.213(-0.679,0.250) | 0.495(-0.097,1.098) |
| Mean Age | 0.056(-0.529,-0.651) | 0.105(-0.331,0.570) | -0.149(-0.759,0.451) |
| Exercise Frequency | **-0.548(-1.052,-0.027)*** | -0.300(-0.790,0.197) | -0.493(-1.119,0.119) |
| Exercise Period | -0.112(-0.671,0.404) | -0.403(-0.871,0.062) | -0.339(-1.021,0.325) |
| Time of Single Session | -0.049(-0.551,0.634) | 0.014(-0.514,0.569) | -0.034(-0.719,0.648) |

*Crl*: credible interval; *: significant influence factors, 95% Crl does not contain zero.

## 6.2 FBG

## 6.2.1 Publication year

When the model was adjusted for centering value of publish year 2015, the hierarchy from the unadjusted model retained.


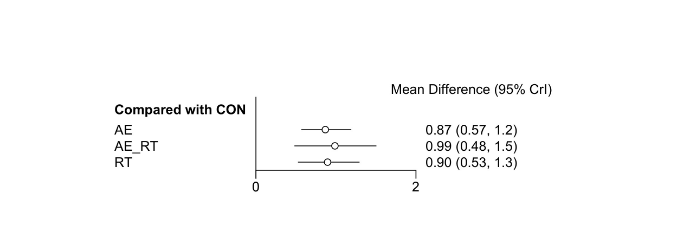


**Figure 6.2.1** Forest plot overall change in general symptoms adjusted for publish year 2015. Exercise type are ranked according to MD compared to CON. Treatments crossing the y-axis are not significantly different from CON. *MD*: Mean Difference, *AE*: Aerobic exercise, *RT*: Resistance training, *AE+RT*: Aerobic exercise combined with resistance training, *Con*: No exercise.

## 6.2.2 Sample size

When the model was adjusted for centering value of sample size 75, the hierarchy from the unadjusted model retained.


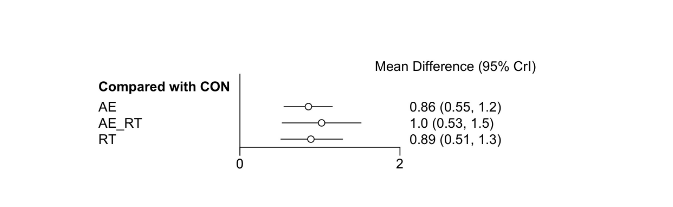


**Figure 6.2.2** Forest plot overall change in general symptoms adjusted for sample size 75. Exercise type are ranked according to MD compared to CON. Treatments crossing the y-axis are not significantly different from CON. *MD*: Mean Difference, *AE*: Aerobic exercise, *RT*: Resistance training, *AE+RT*: Aerobic exercise combined with resistance training, *Con*: No exercise.

## 6.2.3 Percentage of Male

When the model was adjusted for centering value of male’s percentage 37%, hierarchy from the unadjusted model retained.


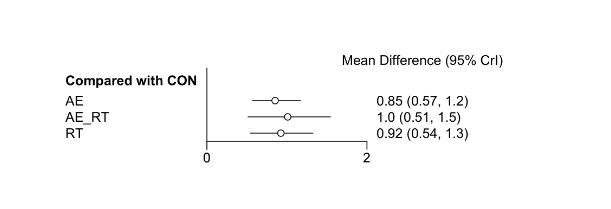


**Figure 6.2.3** Forest plot overall change in general symptoms adjusted for male’s percentage 37%. Exercise type are ranked according to MD compared to CON. Treatments crossing the y-axis are not significantly different from CON. *MD*: Mean Difference, *AE*: Aerobic exercise, *RT*: Resistance training, *AE+RT*: Aerobic exercise combined with resistance training, *Con*: No exercise.

## 6.2.4 Mean age

When the model was adjusted for centering value of mean age 54, the hierarchy from the unadjusted model retained.

`
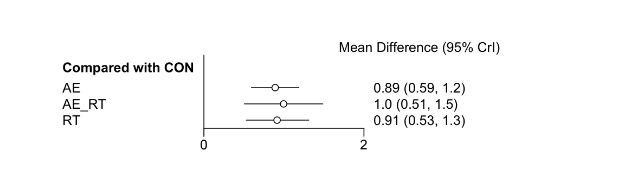


**Figure 6.2.4** Forest plot overall change in general symptoms adjusted for mean age 54. Exercise type are ranked according to MD compared to CON. Treatments crossing the y-axis are not significantly different from CON. *MD*: Mean Difference, *AE*: Aerobic exercise, *RT*: Resistance training, *AE+RT*: Aerobic exercise combined with resistance training, *Con*: No exercise.

## 6.2.5 Exercise frequency

When the model was adjusted for centering value of exercise frequency 3(times/week), the hierarchy from the unadjusted model retained.


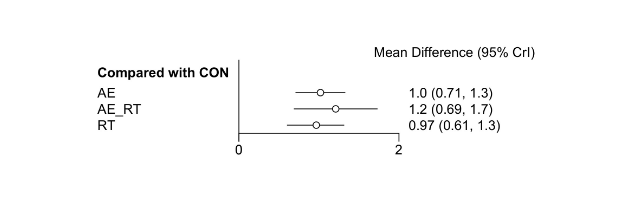


**Figure 6.2.5** Forest plot overall change in general symptoms adjusted for exercise frequency 3(times/week). Exercise type are ranked according to MD compared to CON. Treatments crossing the y-axis are not significantly different from CON. *MD*: Mean Difference, *AE*: Aerobic exercise, *RT*: Resistance training, *AE+RT*: Aerobic exercise combined with resistance training, *Con*: No exercise.

## 6.2.6 Exercise period

When the model was adjusted for exercise period 37(weeks), the hierarchy from the unadjusted model retained.


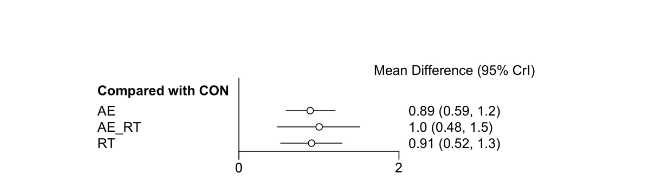


**Figure 6.2.6** Forest plot overall change in general symptoms adjusted for exercise period 37(weeks). Exercise type are ranked according to MD compared to CON. Treatments crossing the y-axis are not significantly different from CON. *MD*: Mean Difference, *AE*: Aerobic exercise, *RT*: Resistance training, *AE+RT*: Aerobic exercise combined with resistance training, *Con*: No exercise.

## 6.2.7 Time of single session

When the model was adjusted for centering value of time of single session 49 minutes, the hierarchy from the unadjusted model retained.


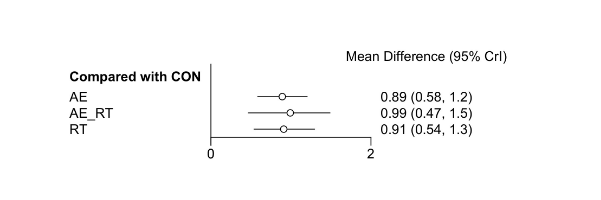


**Figure 6.2.7** Forest plot overall change in general symptoms adjusted for time of single session 49 minutes. Exercise type are ranked according to MD compared to CON. Treatments crossing the y-axis are not significantly different from CON. *MD*: Mean Difference, *AE*: Aerobic exercise, *RT*: Resistance training, *AE+RT*: Aerobic exercise combined with resistance training, *Con*: No exercise.

## 6.3 2hPG

## 6.3.1 Publication year

When the model was adjusted for centering value of publish year 2017, the hierarchy from the unadjusted model retained.


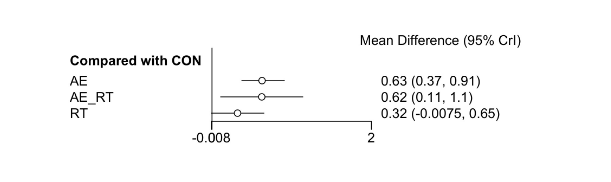


**Figure 6.3.1** Forest plot overall change in general symptoms adjusted for publish year 2017. Exercise type are ranked according to MD compared to CON. Treatments crossing the y-axis are not significantly different from CON. *MD*: Mean Difference, *AE*: Aerobic exercise, *RT*: Resistance training, *AE+RT*: Aerobic exercise combined with resistance training, *Con*: No exercise.

## 6.3.2 Sample size

When the model was adjusted for centering value of sample size 90, hierarchy from the unadjusted model retained


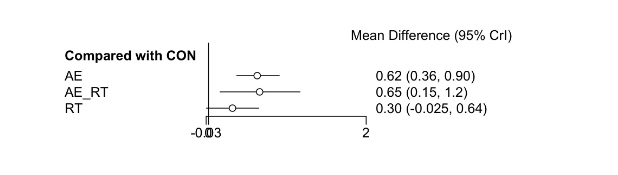


**Figure 6.3.2** Forest plot overall change in general symptoms adjusted for sample size 90. Exercise type are ranked according to MD compared to CON. Treatments crossing the y-axis are not significantly different from CON. *MD*: Mean Difference, *AE*: Aerobic exercise, *RT*: Resistance training, *AE+RT*: Aerobic exercise combined with resistance training, *Con*: No exercise.

## 6.3.3 Percentage of male

When the model was adjusted for centering value of male’s percentage 52%, the hierarchy from the unadjusted model retained.


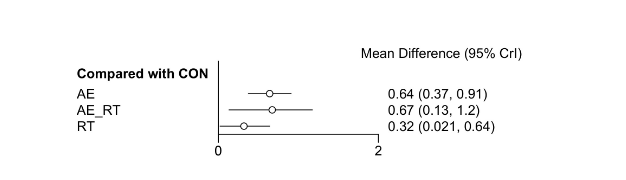


**Figure 6.3.3** Forest plot overall change in general symptoms adjusted for male’s percentage 52%. Exercise type are ranked according to MD compared to CON. Treatments crossing the y-axis are not significantly different from CON. *MD*: Mean Difference, *AE*: Aerobic exercise, *RT*: Resistance training, *AE+RT*: Aerobic exercise combined with resistance training, *Con*: No exercise.

## 6.3.4 Mean age

When the model was adjusted for centering value of mean age 57, the hierarchy from the unadjusted model retained.


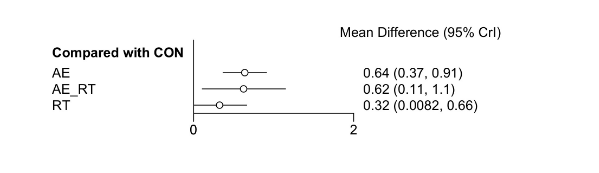


**Figure 6.3.4** Forest plot overall change in general symptoms adjusted for mean age 57. Exercise type are ranked according to MD compared to CON. Treatments crossing the y-axis are not significantly different from CON. *MD*: Mean Difference, *AE*: Aerobic exercise, *RT*: Resistance training, *AE+RT*: Aerobic exercise combined with resistance training, *Con*: No exercise.

## 6.3.5 Exercise frequency

When the model was adjusted for centering value of exercise frequency 3(times/week), the hierarchy from the unadjusted model retained.


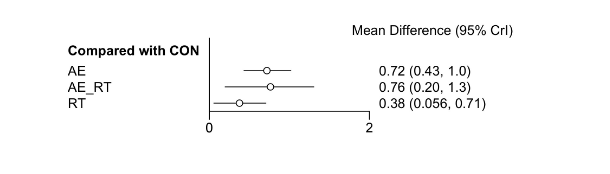


**Figure 6.3.5** Forest plot overall change in general symptoms adjusted for exercise frequency 3(times/week). Exercise type are ranked according to MD compared to CON. Treatments crossing the y-axis are not significantly different from CON. *MD*: Mean Difference, *AE*: Aerobic exercise, *RT*: Resistance training, *AE+RT*: Aerobic exercise combined with resistance training, *Con*: No exercise.

## 6.3.6 Exercise period

When the model was adjusted for exercise period 45(weeks), the hierarchy from the unadjusted model retained.


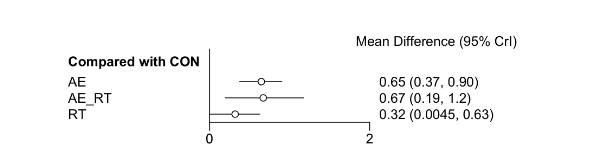


**Figure 6.3.6** Forest plot overall change in general symptoms adjusted for exercise period 45(weeks). Exercise type are ranked according to MD compared to CON. Treatments crossing the y-axis are not significantly different from CON. *MD*: Mean Difference, *AE*: Aerobic exercise, *RT*: Resistance training, *AE+RT*: Aerobic exercise combined with resistance training, *Con*: No exercise.

## 6.3.7 Time of single session

When the model was adjusted for centering value of time of single session 52 minutes, the hierarchy from the unadjusted model retained.


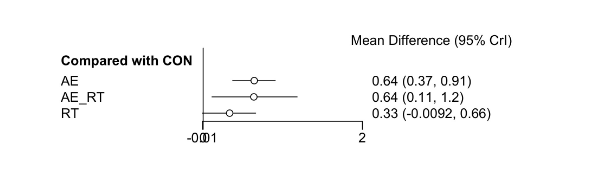


**Figure 6.3.7** Forest plot overall change in general symptoms adjusted for time of single session 52 minutes. Exercise type are ranked according to MD compared to CON. Treatments crossing the y-axis are not significantly different from CON. *MD*: Mean Difference, *AE*: Aerobic exercise, *RT*: Resistance training, *AE+RT*: Aerobic exercise combined with resistance training, *Con*: No exercise.

## 6.4 HbA1c

## 6.4.1 Publication year

When the model was adjusted for centering value of publish year 2017, the hierarchy from the unadjusted model retained.


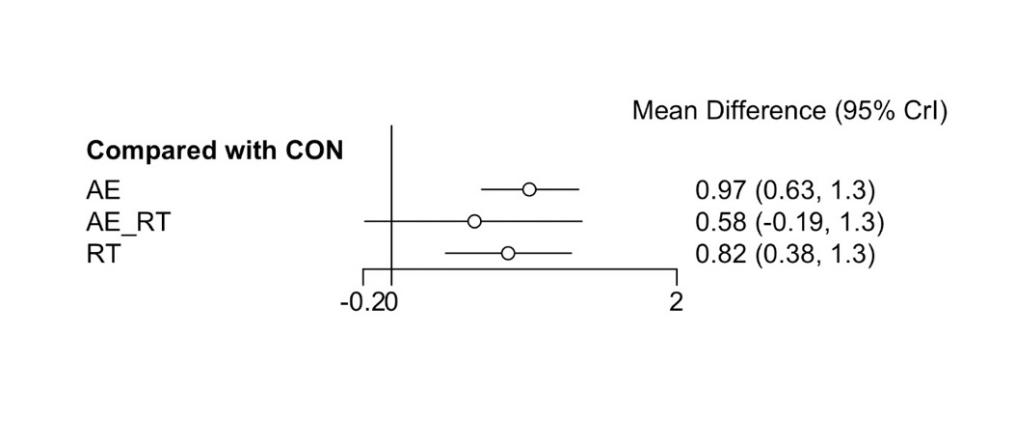


**Figure 6.4.1** Forest plot overall change in general symptoms adjusted for publish year 2017. Exercise type are ranked according to MD compared to CON. Treatments crossing the y-axis are not significantly different from CON. *MD*: Mean Difference, *AE*: Aerobic exercise, *RT*: Resistance training, *AE+RT*: Aerobic exercise combined with resistance training, *Con*: No exercise.

## 6.4.2 Sample size

When the model was adjusted for centering value of sample size 97, hierarchy from the unadjusted model retained


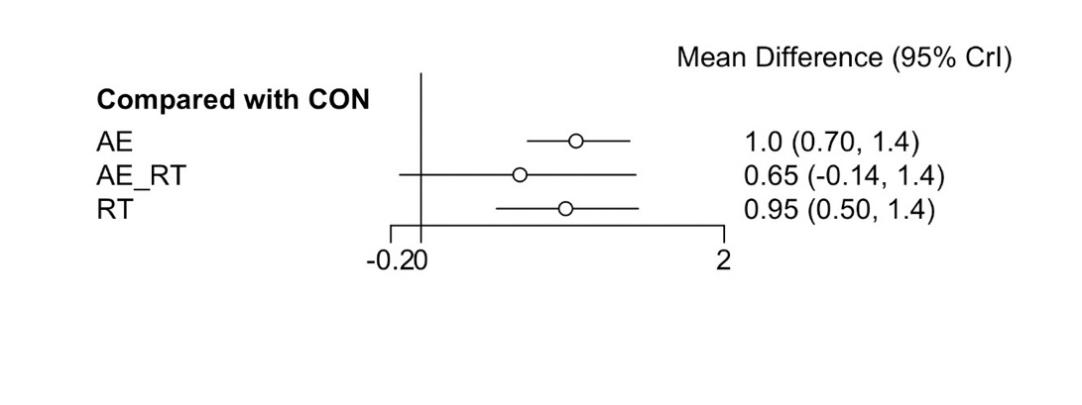


**Figure 6.4.2** Forest plot overall change in general symptoms adjusted for sample size 97. Exercise type are ranked according to MD compared to CON. Treatments crossing the y-axis are not significantly different from CON. *MD*: Mean Difference, *AE*: Aerobic exercise, *RT*: Resistance training, *AE+RT*: Aerobic exercise combined with resistance training, *Con*: No exercise.

## 6.4.3 Percentage of male

When the model was adjusted for centering value of male’s percentage 51%, the hierarchy from the unadjusted model retained.


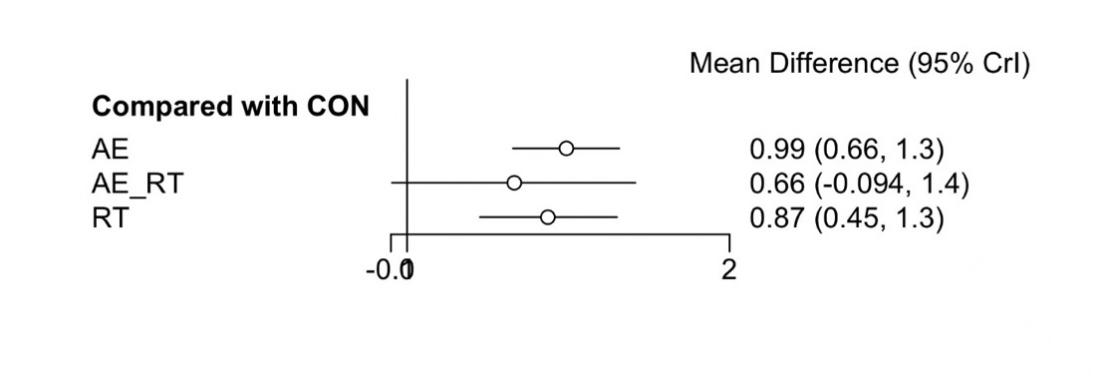


**Figure 6.4.3** Forest plot overall change in general symptoms adjusted for male’s percentage 51%. Exercise type are ranked according to MD compared to CON. Treatments crossing the y-axis are not significantly different from CON. *MD*: Mean Difference, *AE*: Aerobic exercise, *RT*: Resistance training, *AE+RT*: Aerobic exercise combined with resistance training, *Con*: No exercise.

## 6.4.4 Mean age

When the model was adjusted for centering value of mean age 57, the hierarchy from the unadjusted model retained.


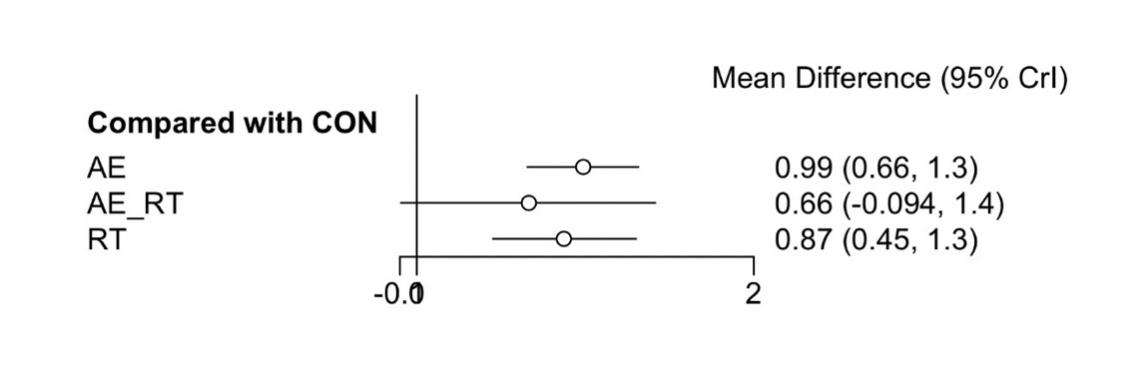


**Figure 6.4.4** Forest plot overall change in general symptoms adjusted for mean age 57. Exercise type are ranked according to MD compared to CON. Treatments crossing the y-axis are not significantly different from CON. *MD*: Mean Difference, *AE*: Aerobic exercise, *RT*: Resistance training, *AE+RT*: Aerobic exercise combined with resistance training, *Con*: No exercise.

## 6.4.5 Exercise frequency

When the model was adjusted for centering value of exercise frequency 3(times/week), the hierarchy from the unadjusted model retained.


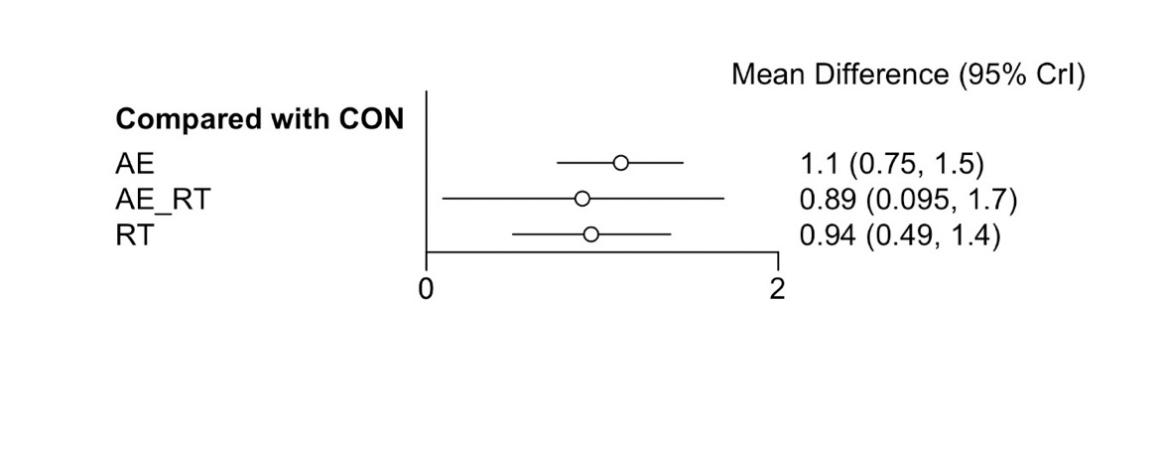


**Figure 6.4.5** Forest plot overall change in general symptoms adjusted for exercise frequency 3(times/week). Exercise type are ranked according to MD compared to CON. Treatments crossing the y-axis are not significantly different from CON. *MD*: Mean Difference, *AE*: Aerobic exercise, *RT*: Resistance training, *AE+RT*: Aerobic exercise combined with resistance training, *Con*: No exercise.

## 6.4.6 Exercise period

When the model was adjusted for exercise period 48(weeks), the hierarchy from the unadjusted model retained.


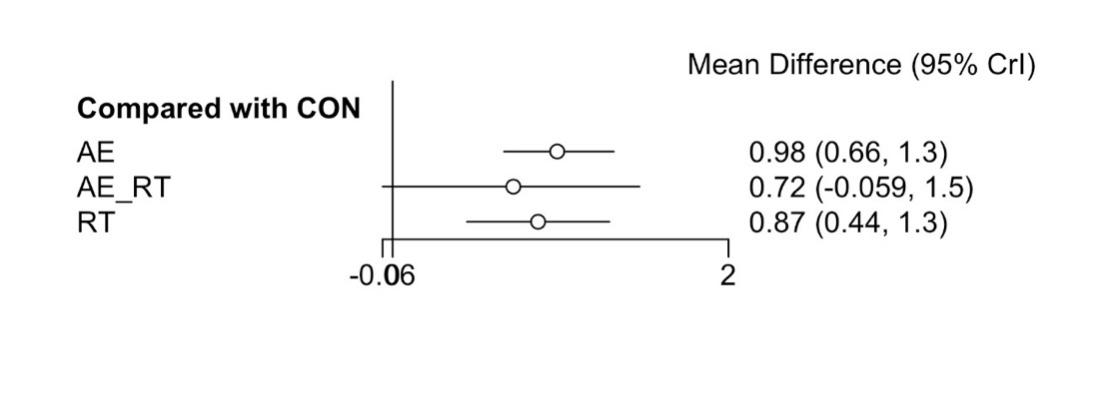


**Figure 6.4.6** Forest plot overall change in general symptoms adjusted for exercise period 48(weeks). Exercise type are ranked according to MD compared to CON. Treatments crossing the y-axis are not significantly different from CON. *MD*: Mean Difference, *AE*: Aerobic exercise, *RT*: Resistance training, *AE+RT*: Aerobic exercise combined with resistance training, *Con*: No exercise.

## 6.4.7 Time of single session

When the model was adjusted for centering value of time of single session 50 minutes, the hierarchy from the unadjusted model retained.


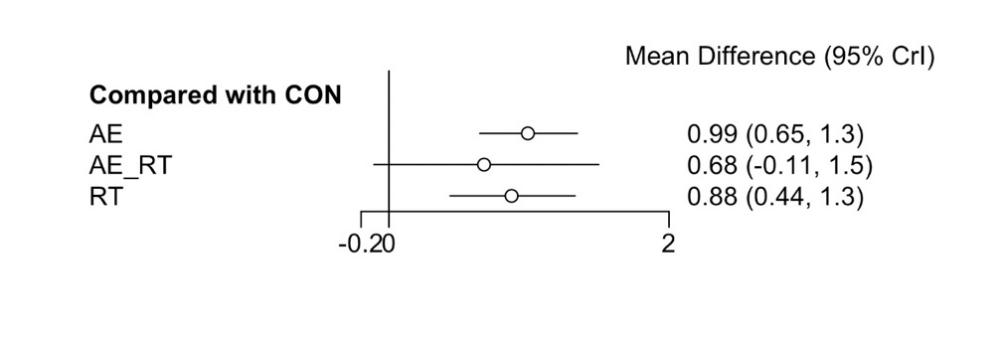


**Figure 6.4.7** Forest plot overall change in general symptoms adjusted for time of single session 50 minutes. Exercise type are ranked according to MD compared to CON. Treatments crossing the y-axis are not significantly different from CON. *MD*:Mean Difference, *AE*: Aerobic exercise, *RT*: Resistance training, *AE+RT*: Aerobic exercise combined with resistance training, *Con*: No exercise.

## 6.5 subgroups analysis

**6.5.1 FBG**

## 6.5.1.1 Exercise Frequency < 3


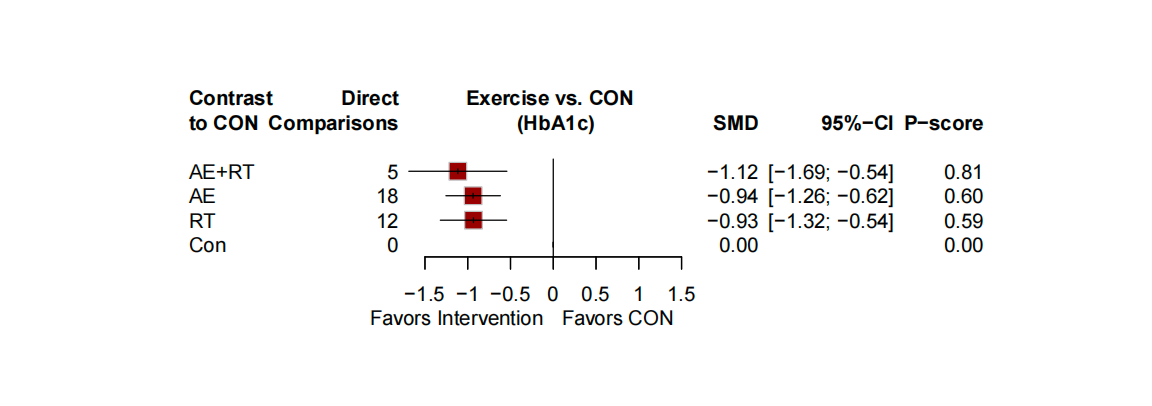


**Figure 6.5.1.1** subgroups analysis of exercise frequency < 3. S*MD*: Standardized Mean Difference, *AE*: Aerobic exercise, *RT*: Resistance training, *AE+RT*: Aerobic exercise combined with resistance training, *Con*: No exercise.

## 6.5.1.2 Exercise Frequency ≥ 3

##
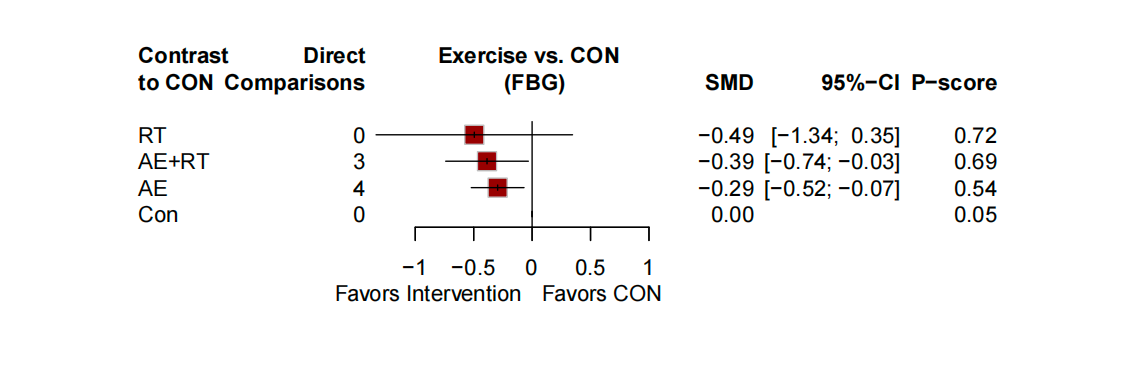


**Figure 6.5.1.2** subgroups analysis of exercise frequency ≥ 3. S*MD*: Standardized Mean Difference, *AE*: Aerobic exercise, *RT*: Resistance training, *AE+RT*: Aerobic exercise combined with resistance training, *Con*: No exercise.

**Supplementary 7: Evaluation of inconsistency**

**Table 7.1 Details of SIDE splitting results**

| Comparison | k | prop | NMA | |  | direct | |  | indir | |  | diff | | p |
| --- | --- | --- | --- | --- | --- | --- | --- | --- | --- | --- | --- | --- | --- | --- |
|  |  |  | TE | seTE |  | TE | seTE |  | TE | seTE |  | TE | seTE |  |
| **FBG** | | | | | | | | | | | | | | |
| AE vs AE+RT | 3 | 0.39 | 0.0429557 | 0.09998562 |  | 0.0209434 | 0.16069276 |  | 0.0568614 | 0.12772075 |  | -0.0359178 | 0.20526752 | 0.8611 |
| AE vs Con | 20 | 0.90 | -0.4011260 | 0.05729967 |  | -0.403191 | 0.06036294 |  | -0.3823149 | 0.18218411 |  | -0.02087615 | 0.19192377 | 0.9134 |
| AE vs RT | 9 | 0.74 | 0.0275212 | 0.07778403 |  | 0.0389447 | 0.09058668 |  | 0.1517650 | -0.3019965 |  | 0.04348729 | 0.17674438 | 0.8056 |
| AE+RT vs Con | 6 | 0.71 | -0.4440817 | 0.09226659 |  | -0.419320 | 0.10925892 |  | 0.172270 | -0.8432816 |  | 0.08631786 | 0.203996311 | 0.6722 |
| AE+RT vs RT | 2 | 0.28 | -0.0154344 | 0.10951836 |  | -0.106342 | 0.20779249 |  | 0.1288701 | -0.2330501 |  | -0.12587499 | 0.244510567 | 0.6067 |
| RT vs Con | 12 | 0.78 | -0.4286477 | 0.07420334 |  | -0.456028 | 0.08400902 |  | 0.1582670 | -0.6416634 |  | -0.12456281 | 0.179181362 | 0.4869 |
| **2hPG** | | | | | | | | | | | | | | |
| AE vs AE+RT | 2 | 0.57 | -0.0345307 | 0.26142730 |  | 0.2478737 | 0.34585511 |  | -0.4109722 | 0.39930709 |  | 0.6588460 | 0.52826311 | 0.2123 |
| AE vs Con | 16 | 0.93 | -0.7075446 | 0.13218033 |  | -0.7577326 | 0.13698182 |  | -0.0290556 | 0.50365675 |  | -0.7286769 | 0.52195223 | 0.1627 |
| AE vs RT | 8 | 0.81 | -0.3299032 | 0.16316892 |  | -0.3183679 | 0.18125656 |  | -0.3792008 | 0.37470771 |  | 0.68871769 | 0.41624489 | 0.8838 |
| AE+RT vs Con | 3 | 0.65 | -0.6730138 | 0.25522475 |  | -0.4781696 | 0.31637998 |  | -1.0360949 | 0.43188394 |  | 0.55792534 | 0.53536906 | 0.2974 |
| AE+RT vs RT | 1 | 0.27 | -0.2953725 | 0.27889146 |  | -0.48 | 0.53624101 |  | -0.2269158 | 0.32652726 |  | -0.25308414 | 0.62783315 | 0.6869 |
| RT vs Con | 10 | 0.82 | -0.3776414 | 0.15946476 |  | -0.4050831 | 0.17629668 |  | -0.2541672 | 0.37396122 |  | -0.15091581 | 0.41343381 | 0.7151 |
| **HbA1c** | | | | | | | | | | | | | | |
| AE vs AE+RT | 1 | 0.40 | -0.0233296 | 0.09604994 |  | 0.01 | 0.15136255 |  | -0.0457983 | 0.12427754 |  | 0.05579837 | 0.19584568 | 0.7757 |
| AE vs Con | 17 | 0.95 | -0.2952163 | 0.03590746 |  | -0.2870058 | 0.03677464 |  | -0.4631754 | 0.16632817 |  | 0.17616963 | 0.17034504 | 0.3010 |
| AE vs RT | 8 | 0.85 | -0.0291343 | 0.04670529 |  | -0.0623762 | 0.05077299 |  | 0.1537432 | 0.11908868 |  | -0.2161194 | 0.12946046 | 0.0950 |
| AE+RT vs Con | 2 | 0.54 | -0.2718867 | 0.09434326 |  | -0.3389602 | 0.12878825 |  | -0.1942108 | 0.13859375 |  | -0.1447494 | 0.18919472 | 0.4442 |
| AE+RT vs RT | 1 | 0.38 | -0.0058047 | 0.09906469 |  | 0.2 | 0.16027933 |  | -0.1330265 | 0.12601744 |  | 0.33302656 | 0.20388688 | 0.1024 |
| RT vs Con | 8 | 0.80 | -0.2660819 | 0.04684359 |  | -0.2630430 | 0.05228830 |  | -0.2784367 | 0.10542921 |  | 0.01539365 | 0.11768341 | 0.8959 |

*NA* not available, *k* Number of studies providing direct evidence, *prop* Direct evidence proportion, *nma* Estimated treatment effect (SMD) in network meta-analysis, *direct* Estimated treatment effect (SMD) derived from direct evidence, *indir.* Estimated treatment effect (SMD) derived from indirect evidence, *Diff* Difference between direct and indirect treatment estimates, *p* p-value of test for disagreement (direct versus indirect).

# Supplementary 8: Publication bias

**8.1 FBG**

As shown in the figure below, the funnel plot had good symmetry, and the linear fitting line (green) is not perpendicular to the 0 quadrant, and the result of Egger test showed the p=0.139. Therefore, no small study effect was found for the primary outcome.

**Figure 8.1** The funnel plot of change of Fasting blood glucose. *AE* Aerobic Exercise, *RT* Resistance Training, *AE+RT* Aerobic exercise combined with resistance training, *Con* Control Group.

**8.2 2hPG**

As shown in the figure below, the funnel plot had good symmetry, and the linear fitting line (green) is not perpendicular to the 0 quadrant, and the result of Egger test showed the p=0.586. Therefore, no small study effect was found for the primary outcome.

**Figure 8.2** The funnel plot of change of 2-hour postprandial blood glucose. *AE* Aerobic Exercise, *RT* Resistance Training, *AE+RT* Aerobic exercise combined with resistance training, *Con* Control Group.

**8.3 HbA1c**

As shown in the figure below, the funnel plot had good symmetry, and the linear fitting line (green) is not perpendicular to the 0 quadrant, and the result of Egger test showed the p=0.167. Therefore, no small study effect was found for the primary outcome.

**Figure 8.3** The funnel plot of change of glycosylated hemoglobin. *AE* Aerobic Exercise, *RT* Resistance Training, *AE+RT* Aerobic exercise combined with resistance training, *Con* Control Group.

Supplementary 9: Grading the evidence for depression symptoms of the network meta-analysis using CINeMA

## 9.1 Summary of study limitations of the included studies


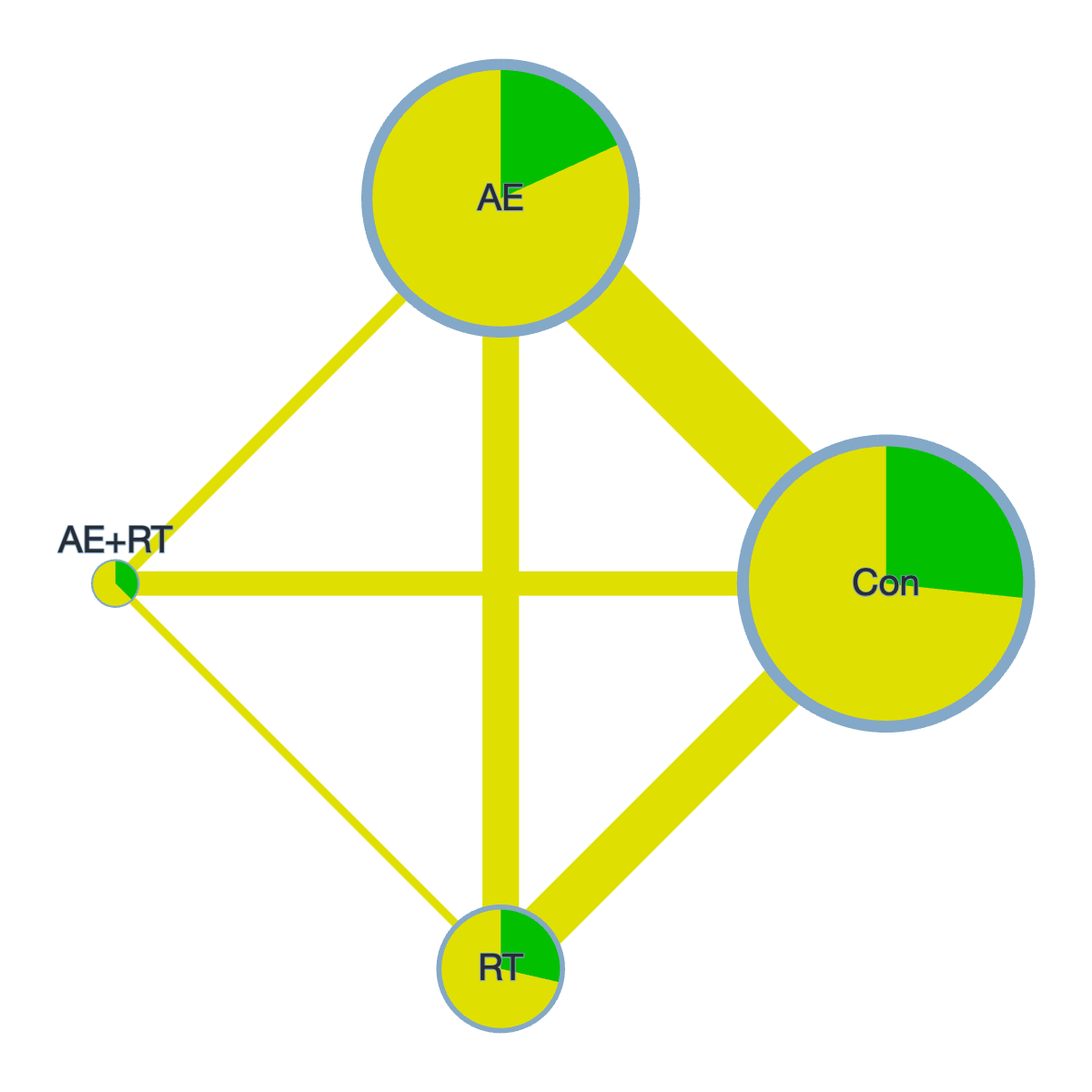


(FBG)


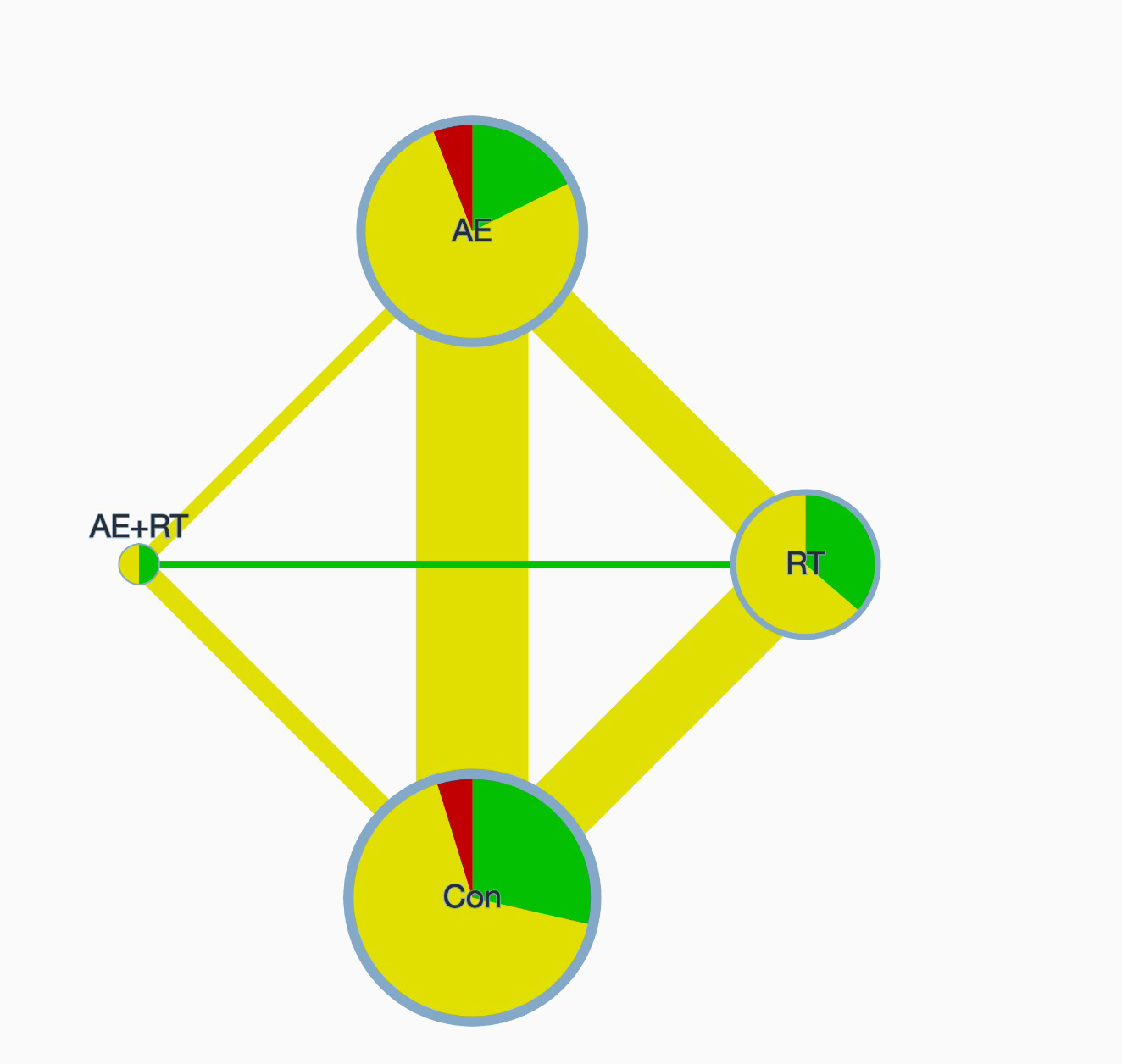


(2hPG)


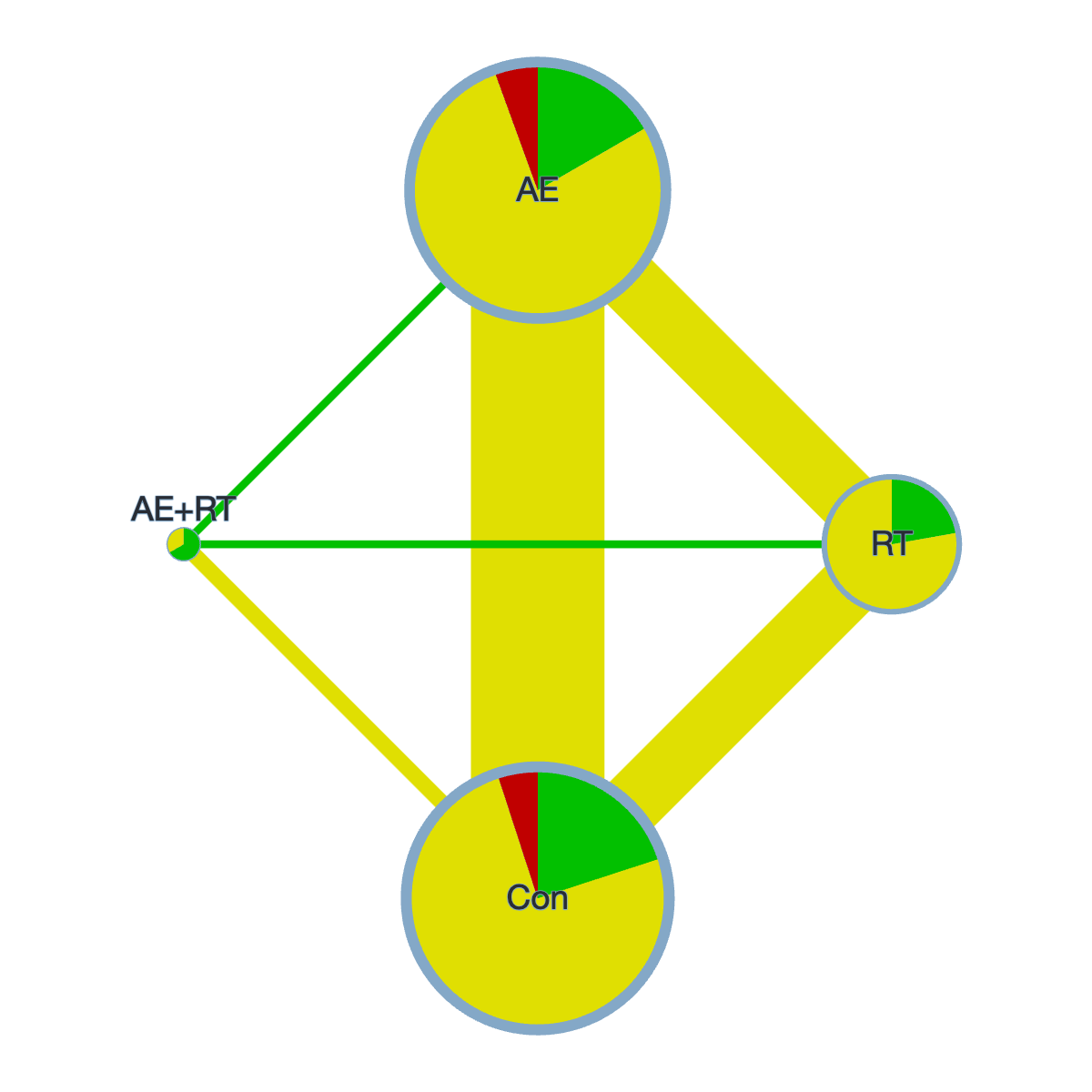


(HbA1c)

**Figure 9.1** Network plot of study limitations of the included studies. Node size by equal size, node color by RoB. The colors in the circles indicate the percentage of low RoB studies (green), moderate RoB studies (yellow), high RoB studies (red) about each physical activity type. Edge width by sample size. Edge color by average RoB. The colors of the lines indicate the summative RoB assessment of each comparison. Low RoB is green, moderate RoB is yellow, high RoB is red. *AE* Aerobic Exercise, *RT* Resistance Training, *AE+RT* Aerobic exercise combined with resistance training, *Con* Control Group.

## 9.2 Reasons for downgrading

Based on the recommendations of the CINeMA online document (https://cinema.ispm.unibe.ch/), we only graded the results of theFasting Blood Glucose(FBG), 2-hour post-load blood glucose(2hPG),Glycosylated hemoglobin(HbA1c), and judged whether each module needs to be downgraded according to the following criteria.

***With-study bias***

We classified the quality evaluation results (Supplementary 3) of each included study into low-risk (PEDro scale ≥6 points), moderate-risk (PDEro scale 4-6 points), and high-risk (PDEro scale <4 points). We selected the rule is average RoB. No need to downgrade when the result was “no concerns”, downgrade one level when “some concerns” and downgrade two level “major concerns”.

***Across-study bias (publication bias)***

Our search was relatively comprehensive, including published and unpublished studies. Due to the language is not limited, Chinese studies were also included in the scope of our inclusion. Even if it is possible that we missed other small unpublished experiments, then it does not seem to affect our results. In Supplementary 8, we evaluated the outcome of publication bias, and comparison-adjusted funnel plots for exercise types showed no evidence of asymmetry. Therefore, the outcome was deemed to have no publication bias.

***Indirectness***

In Supplementary 7, we performed a point inconsistency test on the outcome measures by the SIDE test and found no significant differences. Therefore, no indirectness was assumed and no comparison was downgraded for this reason.

***Imprecision***

The outcome (FBG,2hPG,HbA1c) of this network meta-analysis is a continuous variable, and the effect size measure for continuous outcomes chooses the standardized mean difference (SMD) of the change score (end-point minus baseline score) because the studies use different rating scales or units. Therefore, for CON comparisons the clinically meaningful threshold was set at a standardized mean difference of higher or lower than 0, and for the comparisons of the two types of exercise, the threshold was set at SMD -0.1 and 0.1. If the confidence interval crossed one threshold, it will be downgraded by one level, and two thresholds will be downgraded by two levels.

***Heterogeneity***

For heterogeneity, we used the same threshold as the above clinically significant threshold and follow the recommendations automatically provided by CINeMA (https://cinema.ispm.unibe.ch/). No need to downgrade when the result was “no concerns”, downgrade one level when “some concerns” and downgrade two level when “major concerns”.

***Incoherence***

For incoherence, we will use global and local methods to test the inconsistency of the research results. For global inconsistency, we evaluated inconsistency statistically using the design-by-treatment test. In addition, we will assessment of local inconsistency by separating indirect from direct evidence (SIDE test) using the R netmeta package . No need to downgrade when p >0.1, downgrade one level when p was 0.05-0.1 and downgrade two level when p <0.05 .

***Summarising judgments across the 6 domains***

Τhe final output of CINeMA is a table with the level of concern for each of the 6 domains. we choose to summarise judgments across domains using the 4 levels of confidence of the GRADE approach: very low, low, moderate, or high.(Puhan et al., 2014) Due to factors that may reduce the confidence in a treatment effect may affect more than 1 domain. Indirectness includes consideration of intransitivity, which is manifested as statistical incoherence in the data. Heterogeneity will increase the imprecision of treatment effect, and may be related to the variability of bias within the study or the existence of reporting bias. In addition, in the presence of heterogeneity, the ability to detect important discontinuities will be reduced.(Veroniki, Mavridis, Higgins, & Salanti, 2014) Therefore, the 6 CINeMA domains should be considered jointly rather than in isolation to avoid downgrading the overall level of confidence more than once for related concerns.

## 9.3 CINeMA for the FBG, 2hPG, and HbA1c

| **Comparison** | **Number of studies** | **Within-study bias** | **Reporting bias** | **Indirectness** | **Imprecision** | **Heterogeneity** | **Incoherence** | **Confidence rating** | **Reason(s) for downgrading** |
| --- | --- | --- | --- | --- | --- | --- | --- | --- | --- |
| FBG | | | | | | | | | |
| AE:AE+RT | 3 | Some concerns | Low risk | No concerns | Major concerns | No concerns | No concerns | Very low | [Imprecision] |
| AE:Con | 20 | Some concerns | Low risk | No concerns | No concerns | Major concerns | No concerns | Very low | [] |
| AE:RT | 9 | Some concerns | Low risk | No concerns | Major concerns | No concerns | No concerns | Very low | [] |
| AE+RT:Con | 6 | Some concerns | Low risk | No concerns | No concerns | Major concerns | No concerns | Very low | [] |
| AE+RT:RT | 2 | Some concerns | Low risk | No concerns | Major concerns | No concerns | No concerns | Very low | [] |
| RT:Con | 12 | Some concerns | Low risk | No concerns | No concerns | Major concerns | No concerns | Very low | [] |
| 2hPG | | | | | | | | | |
| AE:AE+RT | 2 | Some concerns | Low risk | No concerns | Major concerns | No concerns | No concerns | Very low | ["Within-study bias","Imprecision"] |
| AE:Con | 16 | Some concerns | Low risk | No concerns | No concerns | Major concerns | No concerns | Very low | [] |
| AE:RT | 8 | Some concerns | Low risk | No concerns | Major concerns | No concerns | No concerns | Very low | [] |
| AE+RT:Con | 3 | Some concerns | Low risk | No concerns | No concerns | Major concerns | No concerns | Very low | [] |
| AE+RT:RT | 1 | Some concerns | Low risk | No concerns | Major concerns | No concerns | No concerns | Very low | [] |
| RT:Con | 10 | Some concerns | Low risk | No concerns | No concerns | Major concerns | No concerns | Very low | [] |
| HbA1c | | | | | | | | | |
| AE:AE+RT | 1 | No concerns | Low risk | No concerns | Major concerns | No concerns | No concerns | Low | [] |
| AE:Con | 17 | Some concerns | Low risk | No concerns | No concerns | Major concerns | No concerns | Very low | [] |
| AE:RT | 8 | Some concerns | Low risk | No concerns | Major concerns | No concerns | No concerns | Very low | [] |
| AE+RT:Con | 2 | No concerns | Low risk | No concerns | No concerns | Major concerns | No concerns | Low | [] |
| AE+RT:RT | 1 | No concerns | Low risk | No concerns | Major concerns | No concerns | No concerns | Low | [] |
| RT:Con | 8 | Some concerns | Low risk | No concerns | No concerns | Major concerns | No concerns | Very low | [] |

*AE* Aerobic Exercise*, RT* Resistance Training*, AE+RT* Aerobic exercise combined with resistance training*, Con* Control Group*.*

**Supplementary 10:** Assessment of Connectivity, Consistency and Transitivity in Network Meta Dose-Response Analysis

**10.1 Connectivity**

Connectivity is a key assumption in network meta-dose analysis, and evidence of unconnectedness may lead to low statistical power and misleading results (Ter Veer, van Oijen, & van Laarhoven, 2019). Our results show that there is no phenomenon of poor connectivity.
**10.1.1 FBG**

**Figures 10.1.1** Treatment-level network. The first value indicates the specific intervention and the second one is the corresponding dose of that intervention. *AE* Aerobic Exercise, *RT* Resistance Training, *AE+RT* Aerobic exercise combined with resistance training.

**10.1.2 2hPG**

**Figures 10.1.2** Treatment-level network. The first value indicates the specific intervention and the second one is the corresponding dose of that intervention. *AE* Aerobic Exercise, *RT* Resistance Training, *AE+RT* Aerobic exercise combined with resistance training.

**10.1.3 HbA1c**

**Figures 10.1.3** Treatment-level network. The first value indicates the specific intervention and the second one is the corresponding dose of that intervention. *AE* Aerobic Exercise, *RT* Resistance Training, *AE+RT* Aerobic exercise combined with resistance training.

**10.2.1 FBG**

**Figure 10.2.1** Agent-level network of FBG. *AE* Aerobic Exercise, *RT* Resistance Training, *AE+RT* Combined aerobic exercise with resistance training.

**10.2.2 2hPG**

**Figure 10.2.2** Agent-level network of 2hPG. *AE* Aerobic Exercise, *RT* Resistance Training, *AE+RT* Combined aerobic exercise with resistance training.

**10.2.3 HbA1c**

**Figure 10.2.3** Agent-level network of HbA1c. *AE* Aerobic Exercise, *RT* Resistance Training, *AE+RT* Combined aerobic exercise with resistance training.

**10.3 Consistency**

We analyzed the data with the consistency model and the unrelated mean effect model, and compared the differences in the deviation, the number of estimated parameters in the network, and the Deviance Informative Criterion (DIC) indicators of the two models. If these are similar, it means that our research has good consistency (Wheeler, Hickson, & Waller, 2010). Comparison of these parameters indicated good consistency across models (Table 10.3).

**Table 10.3** **Consistent and UME models fit comparison.**

**10.3.1 FBG**

| **Model** | **PD** | **Residual deviance** | **Deviance** | **DIC** | **SD** |
| --- | --- | --- | --- | --- | --- |
| Consistent | 64.3 | 68.690 | -127.163 | -63.5 | 0.809 |
| UME | 63.1 | 65.588 | -127.265 | -64.8 | 0.811 |

**10.3.2 2hPG**

| **Model** | **PD** | **Residual deviance** | **Deviance** | **DIC** | **SD** |
| --- | --- | --- | --- | --- | --- |
| Consistent | 46.7 | 48.994 | 0.042 | 46.2 | 0.558 |
| UME | 44.0 | 48.287 | -0.666 | 42.8 | 0.524 |

**10.3.3 HbA1c**

| **Model** | **PD** | **Residual deviance** | **Deviance** | **DIC** | **SD** |
| --- | --- | --- | --- | --- | --- |
| Consistent | 44.8 | 46.352 | -137.047 | -92.8 | 0.813 |
| UME | 43.6 | 47.185 | -136.214 | -93.3 | 0.841 |

PD: Number of estimated parameters; DIC: Deviance Informative Criterion; SD: Standard Deviation; UME: Unrelated Mean Effects. Scientific literature indicated that the main indicator to assess the model fit is the DIC. As lower DIC, better fit.

**10.4 Transitivity**

We assessed transitivity via MBNMA node-splitting approach. This method splits and compares contributions for a particular treatment contrast into direct and indirect evidence (van Valkenhoef, Dias, Ades, & Welton, 2016). Similar effects denote good transitivity. Figures 10.2 and Table 10.3 below present the results for transitivity in this meta-analysis.

**Table 10.4 Node-splitting analysis of inconsistency**

**10.4.1 FBG**

| **Comparison** | **p-value** | **Median** | **2.50%** | **97.50%** |
| --- | --- | --- | --- | --- |
| AE_1250 vs AE_500 | 0.114 |  |  |  |
| -> direct |  | 0.347 | -1.232 | 1.920 |
| -> indirect |  | -0.063 | -0.186 | -0.004 |
| -> MBNMA | | -0.061 | -0.188 | -0.004 |
|  |  |  |  |  |
| AE_750 vs AE_500 | 0.101 |  |  |  |
| -> direct |  | -0.233 | -1.157 | 0.691 |
| -> indirect |  | -0.033 | -0.093 | -0.002 |
| -> MBNMA | | -0.033 | -0.095 | -0.002 |
|  |  |  |  |  |
| RT_1500 vs Placebo_0 | 0.385 |  |  |  |
| -> direct |  | -0.434 | -2.072 | 1.133 |
| -> indirect |  | -1.054 | -1.512 | -0.566 |
| -> MBNMA | | -1.030 | -1.525 | -0.572 |
|  |  |  |  |  |
| RT_1000 vs Placebo_0 | 0.713 |  |  |  |
| -> direct |  | -1.272 | -2.013 | -0.542 |
| -> indirect |  | -1.048 | -1.619 | -0.499 |
| -> MBNMA | | -1.004 | -1.478 | -0.560 |
|  |  |  |  |  |
| RT_750 vs Placebo_0 | 0.737 |  |  |  |
| -> direct |  | -1.113 | -1.696 | -0.502 |
| -> indirect |  | -0.891 | -1.670 | -0.140 |
| -> MBNMA | | -0.983 | -1.439 | -0.548 |
|  |  |  |  |  |
| AE+RT_1500 vs Placebo_0 | 0.515 |  |  |  |
| -> direct |  | -0.552 | -2.225 | 1.072 |
| -> indirect |  | -1.062 | -1.729 | -0.369 |
| -> MBNMA | | -0.974 | -1.581 | -0.374 |
|  |  |  |  |  |
| AE+RT_1250 vs Placebo_0 | 0.550 |  |  |  |
| -> direct |  | -0.574 | -1.987 | 0.809 |
| -> indirect |  | -1.080 | -1.770 | -0.389 |
| -> MBNMA | | -0.964 | -1.568 | -0.373 |
|  |  |  |  |  |
| AE+RT_1000 vs Placebo_0 | 0.596 |  |  |  |
| -> direct |  | -1.089 | -1.850 | -0.328 |
| -> indirect |  | -0.646 | -1.589 | 0.362 |
| -> MBNMA | | -0.952 | -1.543 | -0.363 |
|  |  |  |  |  |
| AE_1750vs Placebo_0 | 0.279 |  |  |  |
| -> direct |  | -0.153 | -1.775 | 1.460 |
| -> indirect |  | -0.924 | -1.301 | -0.550 |
| -> MBNMA | | -0.881 | -1.243 | -0.515 |
|  |  |  |  |  |
| AE_1000 vs Placebo_0 | 0.683 |  |  |  |
| -> direct |  | -0.778 | -1.568 | -0.005 |
| -> indirect |  | -0.875 | -1.274 | -0.459 |
| -> MBNMA | | -0.853 | -1.199 | -0.499 |

**10.4.2 2hPG**

| **Comparison** | **p-value** | **Median** | **2.50%** | **97.50%** |
| --- | --- | --- | --- | --- |
| AE_1250 vs AE_500 | 0.143 |  |  |  |
| -> direct |  | -0.138 | -1.224 | 0.936 |
| -> indirect |  | -0.047 | -0.146 | -0.003 |
| -> MBNMA | | -0.048 | -0.146 | -0.002 |
|  |  |  |  |  |
| AE_750 vs AE_500 | 0.054 |  |  |  |
| -> direct |  | 0.416 | -0.894 | 1.737 |
| -> indirect |  | -0.025 | -0.074 | -0.001 |
| -> MBNMA | | -0.026 | -0.074 | -0.001 |
|  |  |  |  |  |
| RT_1500 vs Placebo_0 | 0.413 |  |  |  |
| -> direct |  | -0.598 | -1.883 | 0.671 |
| -> indirect |  | -0.390 | -0.752 | -0.030 |
| -> MBNMA | | -0.394 | -0.740 | -0.057 |
|  |  |  |  |  |
| RT_1000 vs Placebo_0 | 0.558 |  |  |  |
| -> direct |  | -0.166 | -0.671 | 0.337 |
| -> indirect |  | -0.445 | -0.890 | -0.003 |
| -> MBNMA | | -0.385 | -0.721 | -0.055 |
|  |  |  |  |  |
| RT_750 vs Placebo_0 | 0.595 |  |  |  |
| -> direct |  | -0.557 | -1.014 | -0.105 |
| -> indirect |  | -0.303 | -0.821 | 0.220 |
| -> MBNMA | | -0.375 | -0.708 | -0.053 |
|  |  |  |  |  |
| AE+RT_1500 vs Placebo_0 | 0.511 |  |  |  |
| -> direct |  | -0.292 | -1.563 | 0.879 |
| -> indirect |  | -0.860 | -1.567 | -0.178 |
| -> MBNMA | | -0.707 | -1.283 | -0.108 |
|  |  |  |  |  |
| AE+RT_1000 vs Placebo_0 | 0.736 |  |  |  |
| -> direct |  | -0.644 | -1.362 | 0.094 |
| -> indirect |  | -0.420 | -1.549 | 0.689 |
| -> MBNMA | | -0.688 | -1.258 | -0.106 |
|  |  |  |  |  |
| AE_1750 vs Placebo_0 | 0.386 |  |  |  |
| -> direct |  | -1.003 | -2.250 | 0.236 |
| -> indirect |  | -0.705 | -0.999 | -0.382 |
| -> MBNMA | | -0.723 | -1.018 | -0.415 |
|  |  |  |  |  |
| AE_1000 vs Placebo_0 | 0.680 |  |  |  |
| -> direct |  | -0.585 | -1.142 | -0.021 |
| -> indirect |  | -0.732 | -1.075 | -0.396 |
| -> MBNMA | | -0.698 | -0.977 | -0.407 |
|  |  |  |  |  |
| AE_1000 vs Placebo_0 | 0.291 |  |  |  |
| -> direct |  | -1.109 | -2.209 | -0.036 |
| -> indirect |  | -0.548 | -0.842 | -0.295 |
| -> MBNMA | | -0.577 | -0.853 | -0.325 |

**10.4.3 HbA1c**

| **Comparison** | **p-value** | **Median** | **2.50%** | **97.50%** |
| --- | --- | --- | --- | --- |
| AE_750 vs AE_500 | 0.049 |  |  |  |
| -> direct |  | 0.774 | -0.870 | 2.394 |
| -> indirect |  | -0.038 | -0.108 | -0.002 |
| -> MBNMA | | -0.038 | -0.106 | -0.002 |
|  |  |  |  |  |
| RT_1000 vs AE_0 | 0.175 |  |  |  |
| -> direct |  | -1.696 | -2.520 | -0.957 |
| -> indirect |  | -0.712 | -1.350 | -0.034 |
| -> MBNMA | | -0.940 | -1.467 | -0.401 |
|  |  |  |  |  |
| RT_750 vs Placebo_0 | 0.595 |  |  |  |
| -> direct |  | -0.713 | -1.384 | -0.028 |
| -> indirect |  | -1.090 | -1.819 | -0.408 |
| -> MBNMA | | -0.917 | -1.427 | -0.394 |
|  |  |  |  |  |
| RT_500 vs Placebo_0 | 0.497 |  |  |  |
| -> direct |  | -0.702 | -2.267 | 0.784 |
| -> indirect |  | -0.916 | -1.437 | -0.395 |
| -> MBNMA | | -0.867 | -1.375 | -0.375 |
|  |  |  |  |  |
| AE+RT_1500 vs Placebo_0 | 0.645 |  |  |  |
| -> direct |  | -0.263 | -1.881 | 1.399 |
| -> indirect |  | -0.914 | -2.286 | 0.373 |
| -> MBNMA | | -0.684 | -1.700 | 0.311 |
|  |  |  |  |  |
| AE+RT_1000 vs Placebo_0 | 0.485 |  |  |  |
| -> direct |  | -1.092 | -2.533 | 0.247 |
| -> indirect |  | -0.119 | -1.620 | 1.342 |
| -> MBNMA | | -0.665 | -1.641 | 0.304 |
|  |  |  |  |  |
| AE_RT_1750 vs Placebo_0 | 0.193 |  |  |  |
| -> direct |  | 0.010 | -1.674 | 1.689 |
| -> indirect |  | -1.162 | -1.598 | -0.745 |
| -> MBNMA | | -1.097 | -1.519 | -0.694 |
|  |  |  |  |  |
| AE_1250 vs Placebo_0 | 0.440 |  |  |  |
| -> direct |  | -1.024 | -2.269 | 0.590 |
| -> indirect |  | -1.078 | -1.535 | -0.661 |
| -> MBNMA | | -1.080 | -1.491 | -0.682 |
|  |  |  |  |  |
| AE_1000 vs Placebo_0 | 0.484 |  |  |  |
| -> direct |  | -0.728 | -1.632 | 0.152 |
| -> indirect |  | -1.143 | -1.611 | -0.689 |
| -> MBNMA | | -1.064 | -1.469 | -0.674 |
|  |  |  |  |  |
| AE_250 vs Placebo_0 | 0.423 |  |  |  |
| -> direct |  | -0.875 | -2.505 | 0.663 |
| -> indirect |  | -0.896 | -1.310 | -0.521 |
| -> MBNMA | | -0.892 | -1.280 | -0.534 |


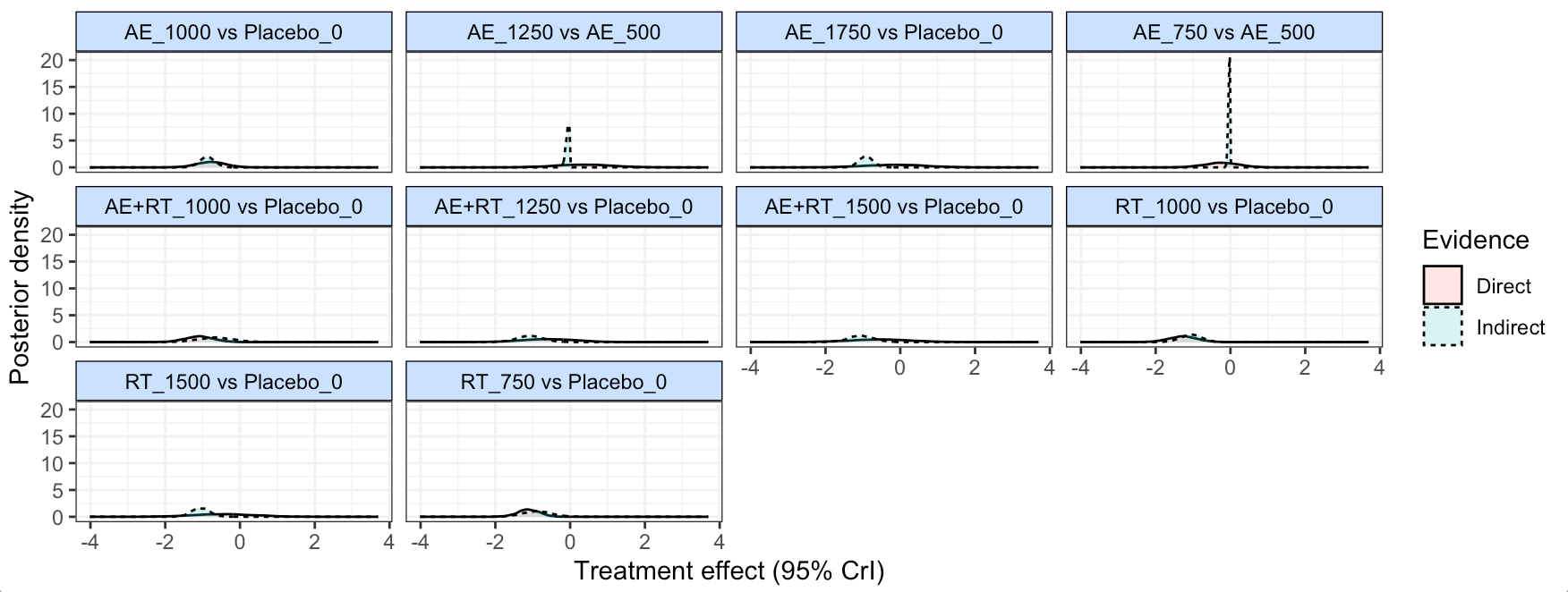


**（FBG）**


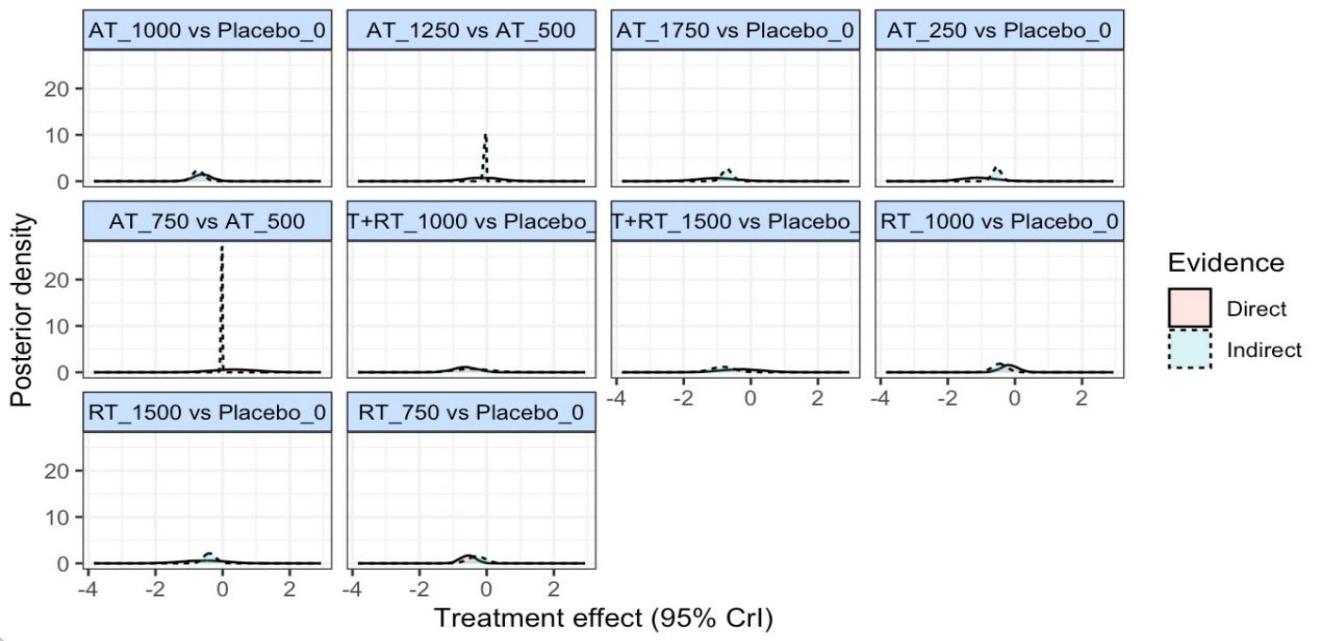


**（2hPG）**


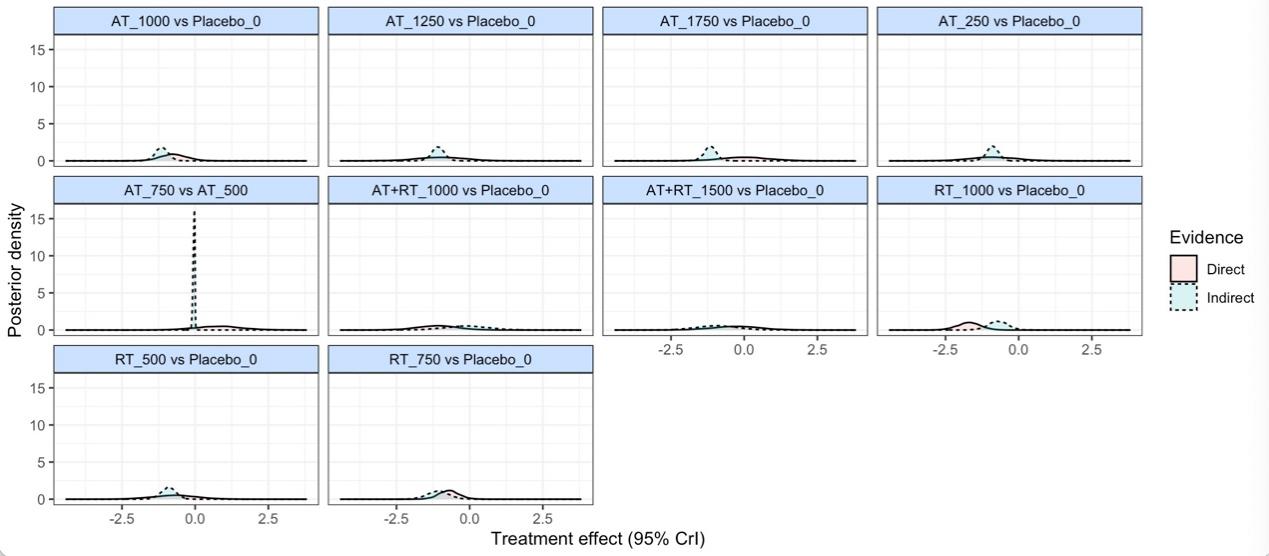


**（HbA1c）**

**Figure 10.5** Node-splitting analysis (density plot). The first value indicates the agent and the second one is the corresponding dose of that agent. *AE* Aerobic Exercise, *RT* Resistance Training, *AE+RT* Combined aerobic exercise with resistance training.

Supplementary 11: Non-linear functions and models fit comparison

The different doses of exercise were meta-analysed as independent and unrelated treatments (i.e., “split” NMA). This step is useful to determine which function fits the data better and should subsequently be used in a Model-Based Network Meta-Analysis (MBNMA) (Pedder, 2021). Figure 11.1 show the different responses (SMD) of each dose for different treatments, respectively.


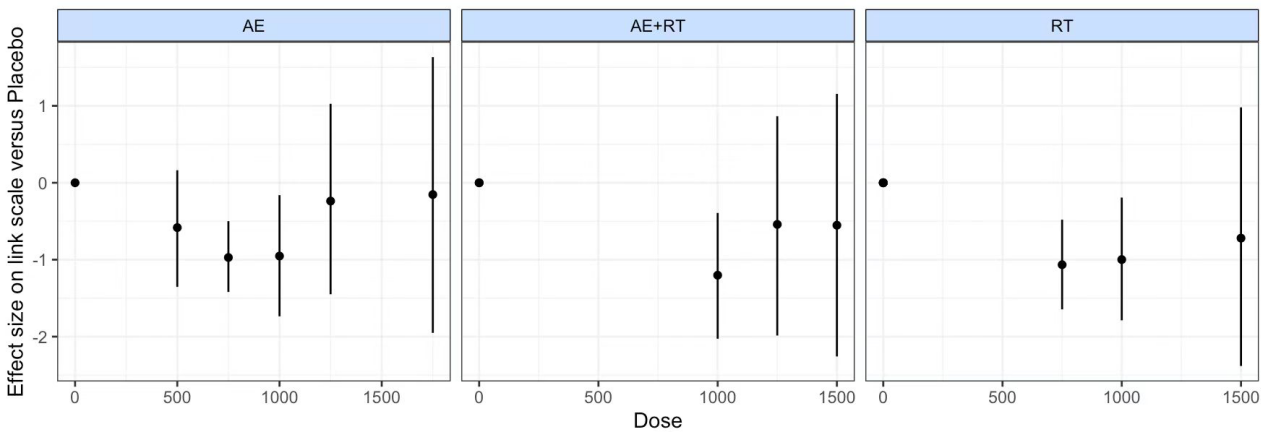
（FBG）


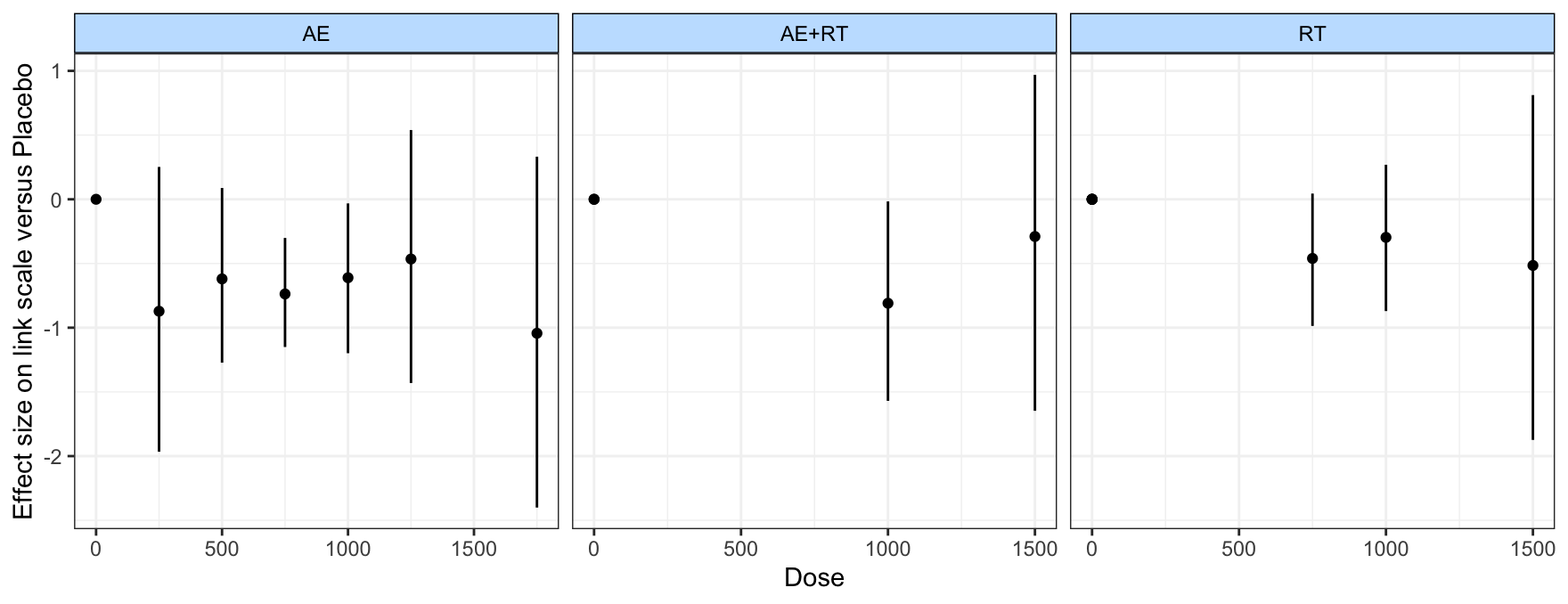


（2hPG）


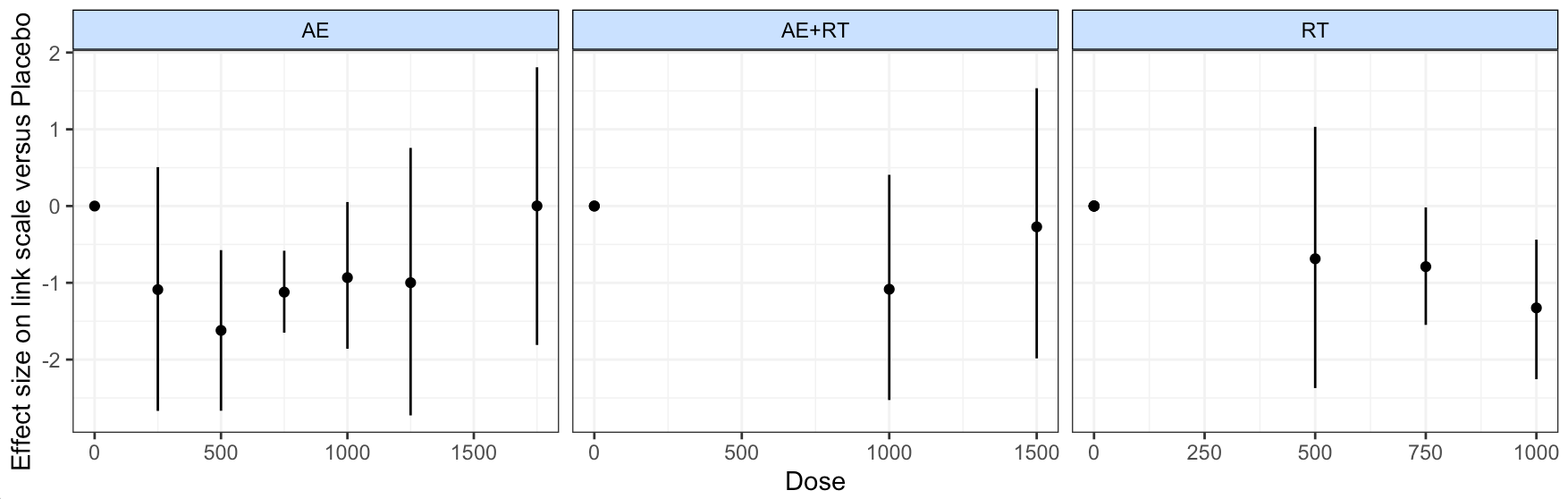


(HbA1c)

**Figure 11.1** “Split” NMA of different exercise agents. *AE* Aerobic Exercise, *RT* Resistance Training, *AE+RT* Combined aerobic exercise with resistance training.

**Table 11.2 shows the fit indices from each of the models fitted. For our data, restricted cubic splines show the best fit and were therefore used in subsequent analyses.**

**Table 11.2 Models fit comparison**

**11.2.1 FBG**

| **Model** | **DIC** | **SD** | **Deviance** | **Residual deviance** | **pD** |
| --- | --- | --- | --- | --- | --- |
| **Emax**  **(****common treatment effects)** | 271.9 | NA | 242.598 | 438.451 | 29.8 |
| **Emax**  **(RANDOM treatment effects)** | -66.2 | 0.742 | -127.588 | 68.265 | 61.9 |
| **Linear**  **(common treatment effects)** | 322.8 | NA | 293.573 | 489.426 | 29.9 |
| **Linear**  **(random treatment effects)** | -64.1 | 0.824 | -127.159 | 68.694 | 63.5 |
| **Exponential**  **(common treatment effects)** | 270.9 | NA | 241.406 | 437.259 | 30.2 |
| **Exponential**  **(RANDOM treatment effects)** | -65.9 | 0.735 | -127.637 | 68.216 | 62.6 |
| **Restricted cubic spline**  **(common treatment effects; 3 knots)** | 261.7 | NA | 229.581 | 425.434 | 32.8 |
| **Restricted cubic spline**  **(random treatment effects; 3 knots)** | -65.3 | 0.741 | 68.281 | -127.572 | 62.8 |
| **Non-parametric monotonically up**  **(common treatment effects)** | 732.7 | NA | 705.218 | 901.071 | 28.3 |
| **Non-parametric monotonically up**  **(RANDOM treatment effects)** | -61.9 | 1.367 | -127.255 | 68.598 | 65.9 |

**11.2.2 2hPG**

| **Model** | **DIC** | **SD** | **Deviance** | **Residual deviance** | **pD** |
| --- | --- | --- | --- | --- | --- |
| **Emax**  **(common treatment effects)** | 166.2 | NA | 144.638 | 193.590 | 22.3 |
| **Emax**  **(RANDOM treatment effects)** | 40.1 | 0.483 | -1.404 | 47.618 | 42.2 |
| **Linear**  **(common treatment effects)** | 192.5 | NA | 171.340 | 220.293 | 22.0 |
| **Linear**  **(random treatment effects)** | 42.8 | 0.535 | -0.930 | 48.023 | 44.2 |
| **Exponential**  **(common treatment effects)** | 164.6 | NA | 143.126 | 192.078 | 22.1 |
| **Exponential**  **(RANDOM treatment effects)** | 40.0 | 0.481 | -1.643 | 47.309 | 42.6 |
| **Restricted cubic spline**  **(common treatment effects; 3 knots)** | 178.1 | NA | 153.485 | 202.438 | 25.1 |
| **Restricted cubic spline**  **(random treatment effects; 3 knots)** | 43.1 | 0.528 | -0.753 | 48.200 | 44.4 |
| **Non-parametric monotonically up**  **(common treatment effects)** | 341.8 | NA | 321.716 | 370.669 | 20.6 |
| **Non-parametric monotonically up**  **(RANDOM treatment effects)** | 48.3 | 0.999 | 1.329 | 50.281 | 47.5 |

**11.2.3 HbA1c**

| **Model** | **DIC** | **SD** | **Deviance** | **Residual deviance** | **pD** |
| --- | --- | --- | --- | --- | --- |
| **Emax**  **(common treatment effects)** | 46.3 | NA | 25.760 | 209.159 | 21.1 |
| **Emax**  **(RANDOM treatment effects)** | -93.6 | 0.737 | -136.293 | 47.246 | 43.5 |
| **Linear**  **(common treatment effects)** | 79.8 | NA | 59.729 | 243.129 | 20.9 |
| **Linear**  **(random treatment effects)** | -92.7 | 0.858 | -136.006 | 47.394 | 44.2 |
| **Exponential**  **(common treatment effects)** | 46.0 | NA | 25.757 | 209.157 | 20.9 |
| **Exponential**  **(RANDOM treatment effects)** | -93.5 | 0.731 | -136.304 | 47.096 | 43.2 |
| **Restricted cubic spline**  **(common treatment effects; 3 knots)** | 43.3 | NA | 20.141 | 203.541 | 23.9 |
| **Restricted cubic spline**  **(random treatment effects; 3 knots)** | -93.7 | 0.741 | -136.820 | 46.580 | 43.8 |
| **Non-parametric monotonically up**  **(common treatment effects)** | 359.7 | NA | 341.288 | 524.688 | 19.1 |
| **Non-parametric monotonically up**  **(RANDOM treatment effects)** | -92.3 | 1.747 | -137.115 | 46.285 | 45.4 |

DIC = Deviance Information Criterion; SD = Between-study Standard Deviation; pD: Number of estimated parameters; NA = Not Applicable. The SD is presented as the main value and (95% Credible Intervals).

Further to model fit indices, deviance plots showing the contribution of each data point to the residual deviance are also useful to confirm the robustness of model selection (Pedder, 2021). Each data point should contribute about 1 to the posterior mean deviance, which indicates good model fit (Dias, Sutton, Ades, & Welton, 2013). The deviance plot for treatment effects (Supplementary Figure 11.3) confirm the robustness of our model selection.


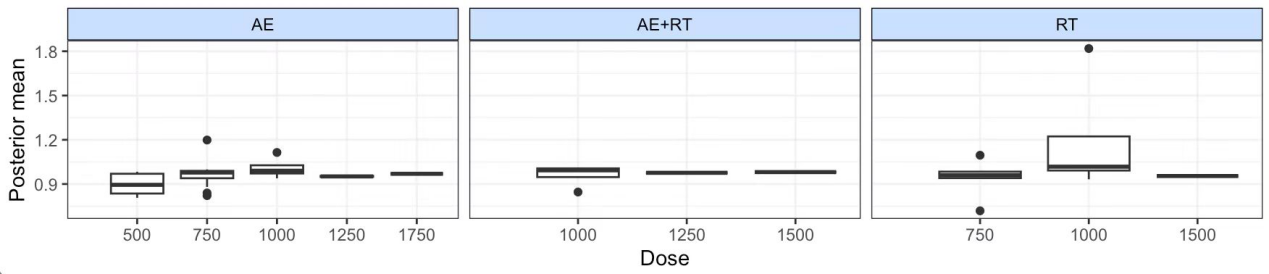
（FBG）


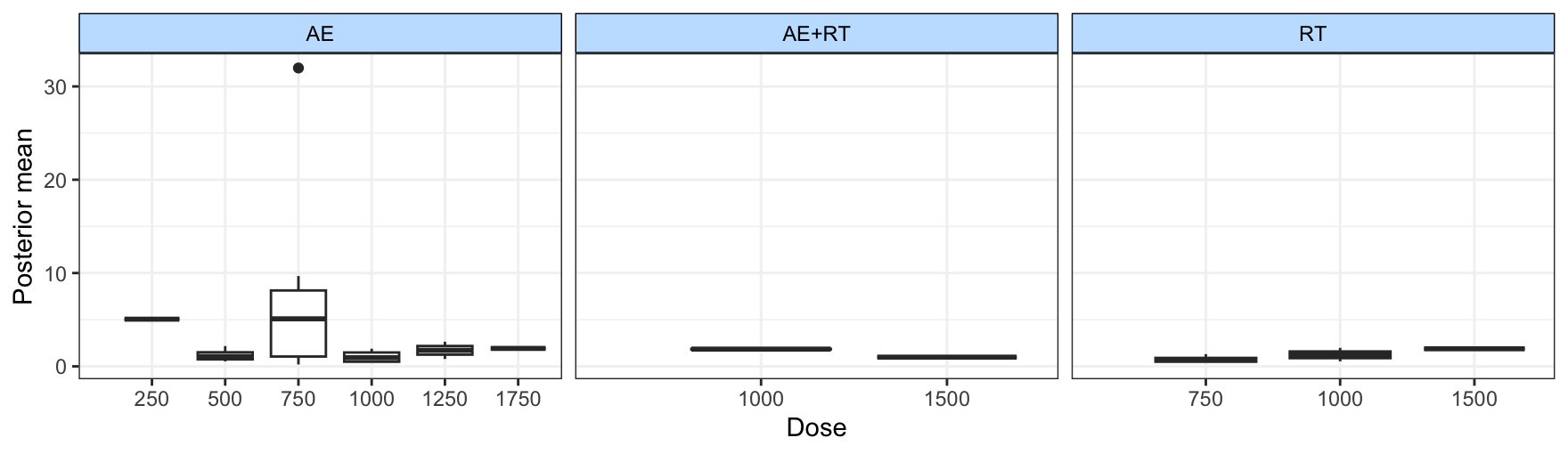
 （2hPG）


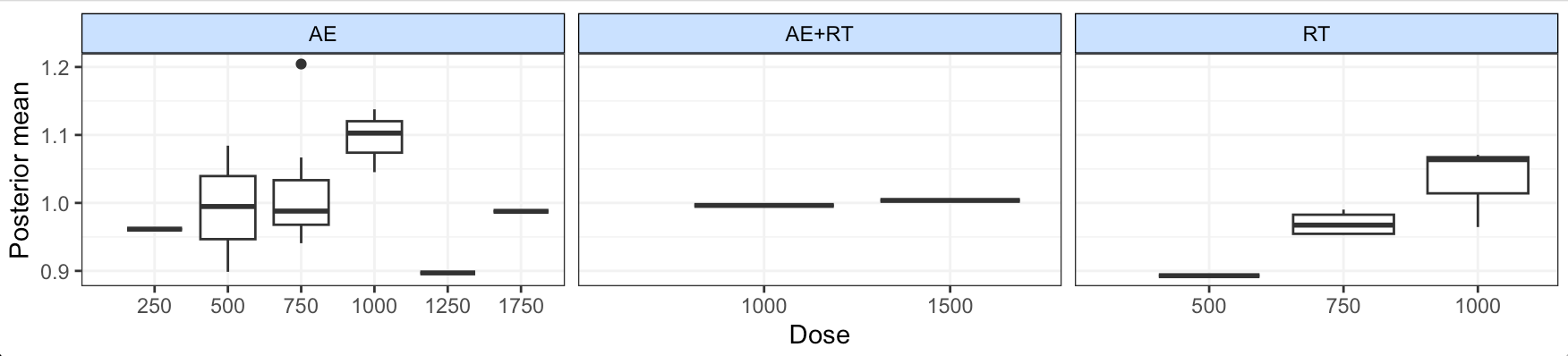
 （HbA1c）

**References**

Dias, S., Sutton, A. J., Ades, A. E., & Welton, N. J. (2013). Evidence synthesis for decision making 2: a generalized linear modeling framework for pairwise and network meta-analysis of randomized controlled trials. *Medical Decision Making : an International Journal of the Society For Medical Decision Making, 33*(5), 607-617. doi:10.1177/0272989X12458724

Pedder, H. (2021). *MBNMAdose: An R package for incorporating dose-response information into Network Meta-Analysis.* Paper presented at the Evidence Synthesis and Meta-Analysis in R Conference 2021.

Puhan, M. A., Schünemann, H. J., Murad, M. H., Li, T., Brignardello-Petersen, R., Singh, J. A., . . . Guyatt, G. H. (2014). A GRADE Working Group approach for rating the quality of treatment effect estimates from network meta-analysis. *BMJ (Clinical research ed.), 349*.

Ter Veer, E., van Oijen, M. G. H., & van Laarhoven, H. W. M. (2019). The Use of (Network) Meta-Analysis in Clinical Oncology. *Frontiers in Oncology, 9*, 822. doi:10.3389/fonc.2019.00822

van Valkenhoef, G., Dias, S., Ades, A. E., & Welton, N. J. (2016). Automated generation of node-splitting models for assessment of inconsistency in network meta-analysis. *Research Synthesis Methods, 7*(1), 80-93. doi:10.1002/jrsm.1167

Veroniki, A. A., Mavridis, D., Higgins, J. P. T., & Salanti, G. (2014). Characteristics of a loop of evidence that affect detection and estimation of inconsistency: a simulation study. *BMC medical research methodology, 14*, 106. doi:10.1186/1471-2288-14-106

Wheeler, D. C., Hickson, D. A., & Waller, L. A. (2010). Assessing Local Model Adequacy in Bayesian Hierarchical Models Using the Partitioned Deviance Information Criterion. *Computational Statistics & Data Analysis, 54*(6), 1657-1671. Retrieved from <https://pubmed.ncbi.nlm.nih.gov/21243121>
